# From forward-backward sweep to backpropagation for optimal control in tumor-immune dynamics

**DOI:** 10.1007/s00285-026-02475-2

**Published:** 2026-10-29

**Authors:** Wenjing Zhang, Wandi Ding, Huaiping Zhu

**Affiliations:** 1 Department of Mathematics and Statistics, Texas Tech University, Lubbock, TX USA; 2 Department of Mathematical Sciences, Middle Tennessee State University, Murfreesboro, TN USA; 3 Department of Mathematics and Statistics, York University, Toronto, ON Canada

**Keywords:** Universal differential equations, Pontryagin’s maximum principle, Forward-backward sweep, Tumor-immune dynamics, Scientific machine learning, 92C50, 49M25, 68T07, 34H05

## Abstract

Optimal control problems in mathematical biology are commonly solved by the forward-backward sweep on the Pontryagin’s Maximum Principle necessary conditions. The sweep has known stability issues at constraint-saturated optima. We frame the same problem within the universal differential equation framework. A neural network represents the control. The cost functional serves as the training loss. We prove that any stationary point of the training loss satisfies the continuous first-order necessary conditions to a discretisation-controlled accuracy. On a grid of bifurcation-parameter and initial-condition scenarios drawn from a tumor-immune dynamics model, the standard sweep fails on most scenarios in the nominal regime. Neural network training reaches a stable optimum on every scenario, with a lower final cost than the converged damped sweep iterate. The pipeline accommodates cost functional variants by changing only the loss function. A Sobol sensitivity analysis confirms robustness of the trained schedule to perturbations of the cost weights.

## Introduction

Optimal control problems, abbreviated OC, in mathematical biology specify a dynamical system, a set of admissible interventions, and a clinical or ecological cost to be minimised. Classical approaches solve them through Pontryagin’s Maximum Principle, abbreviated PMP (Pontryagin 2018), through direct methods (Betts 2010), or through Hamilton-Jacobi-Bellman equations (Bellman 1957). The practitioner standard in mathematical biology is the forward-backward sweep iteration on the PMP necessary conditions (Lenhart and Workman 2007). It is simple to implement. It has known stability issues on stiff systems, on systems near bifurcations, and on problems whose optimum saturates a control constraint (Hairer and Wanner 1996; McAsey et al. 2012).

The forward-backward structure of the sweep mirrors the forward and backward passes of neural network training. Treating the control as a neural network and the cost functional as a training loss recovers the same first-order optimality conditions. The backward pass replaces backward-in-time adjoint integration with machine-precision reverse-mode automatic differentiation through the discretised solver. This idea is the universal differential equation framework, abbreviated UDE (Chen et al. 2018; Rackauckas and Nie 2017; Rackauckas et al. 2020). In this framework a mechanistic model is composed with a neural network that represents the unknown control. Deep learning has previously been applied to optimal control in mathematical biology, including epidemic models (Yin et al. 2023). In the benchmark problems considered here, gradient-based UDE training remained stable in regimes where the classical forward-backward sweep exhibited structural convergence failure.

This paper makes three contributions. First, we prove that any stationary point of the UDE training loss satisfies the continuous PMP first-order necessary conditions to an explicit discretisation-controlled accuracy. Second, we benchmark the UDE approach against the forward-backward sweep on a 4×4 grid of scenarios drawn from a published immune-cancer dynamics model (Zheng and Kim 2021; Zhang et al. 2024). The standard nominal sweep fails on 14 of 16 scenarios by control saturation at the box boundary, whereas UDE training reaches a stable optimum on every scenario and lowers the final cost $J^{*}$ by a factor of 2 to 10 relative to the converged damped sweep iterate. Third, we demonstrate that cost functional variants can be accommodated by changing the loss definition with no change to the training pipeline, on three immunotherapy reformulations. A Sobol global sensitivity analysis on the four cost weights confirms that the qualitative shape of the trained schedule is preserved under perturbation of the cost weights around the baseline.

We position this contribution narrowly. This paper is not a generic neural ordinary differential equation controller, a reinforcement learning method, or a physics-informed neural network for control. It is not a black-box substitution of a neural network for the control variable. It is the identification of a specific structural failure mode of the forward-backward sweep, namely a projection-induced saturated 2-cycle of the iterates. It is a proof that any stationary point of the UDE training loss is consistent with the PMP necessary conditions to a discretisation-controlled accuracy. It is an empirical demonstration of stable training on saturated-optimum regimes where the sweep fails. We do not claim that gradient-based training supersedes classical optimal control methods in general.

The remainder of the paper is organised as follows. Section 2.1 reviews deep neural networks and their training. Section 2.3 introduces the UDE framework. Section 3 proves the correspondence between UDE training and the PMP necessary conditions, including the discretisation-controlled accuracy bound. Section 4 applies the framework to the immune-cancer dynamics benchmark and reports the 4×4 comparison, the multi-formulation experiments, and the sensitivity analysis. Section 5 concludes.

## Background

Section 1 introduced the UDE framework as the structural pairing of a mechanistic differential equation with a neural network for an unknown component. This section reviews the technical machinery needed for Theorem 1 of Sect. 3. Section 2.1 fixes notation for deep neural networks and forward propagation, Sect. 2.2 reviews training by gradient descent with backpropagation, and Sect. 2.3 extends the same machinery to UDEs.

### Deep learning and deep neural network

Deep neural networks (DNNs) consist of an input layer, multiple hidden layers, and an output layer (Sze et al. 2017). Each layer is made up of units, or neurons, which are connected to neurons in the previous and subsequent layers. Each connection is assigned a weight, a parameter that determines the influence of one neuron on another. We now provide a general formulation of DNNs as a foundation for the rest of the paper.

Let $x \in \mathbb {R}^{n_1}$ denote the input vector, where $n_1$ is the dimensionality of the input data. A network with *L* layers has layer 1 being the input layer and layer *L* being the output layer. Each layer *l*, for l=2,3,⋯,L, consists of $n_l$ neurons that perform weighted sums of the inputs from the previous layer, followed by a non-linear activation function. To calculate weighted sums, each layer *l*, for l=2,3,⋯,L, has the matrix of weights, also named as weight matrices and denoted as $W^{(l)} \in \mathbb {R}^{n_{l} \times n_{l-1}}$, and the vector of bias, also named as the bias vector and denoted as $b^{(l)} \in \mathbb {R}^{n_{l}}$, where $n_{l}$ represents the number of neurons in layer *l*, and $n_{l-1}$ represents the number of neurons in the preceding layer. Then, we define the pre-activation $z^{(l)}$ at layer *l*, which is the linear transformation $z^{(l)} = W^{(l)} a^{(l-1)} + b^{(l)}$, where $z^{(l)} =\{z_j^{(l)}\}_{j=1}^{j=n_l} \in \mathbb {R}^{n_{l}}$. Each element $z_j^{(l)}$ denotes the weighted input for neuron *j* at layer *l* with $j=1, \dots , n_l$ (Higham and Higham 2019). The action of the network from neuron *j* at layer *l* results in the output $a^{(l)}_j$. During forward propagation, the relation between the input layer through each hidden layer to the output layer can then be written as

1 $$\begin{aligned} \begin{array}{rll} a^{(1)} & = x & \text {input layer}, \\ z^{(l)} & = W^{(l)} a^{(l-1)} + b^{(l)} & \text {pre-activation at layer }l = 2, 3, \dots , L, \\ a^{(l)} & = \{a_j^{(l)}\}_{j=1}^{j=n_l} = \sigma .\left( z^{(l)} \right) & \text {output or activation at layer }l = 2, 3, \dots , L, \end{array} \end{aligned}$$

where σ is referred to as the nonlinear activation function and l=2,3,⋯,L. The . denotes the element-wise operation, that is, if $x, y \in \mathbb {R}^n$, then $x.y \in \mathbb {R}^n$ is defined by $(x.y)_i = x_i y_i$. Often, the final layer $a^{(L)}$ is an output of a final activation function depending on the task. For regression, the final activation is often taken as the same activation function for the hidden layers. For the purpose of classification, a softmax function, $a_j^{(L)} = e^{z_j^{(L)}}/\sum _{i} e^{z_i^{(L)}}$, may be applied to produce class probabilities. Mathematically, the network can be represented as a series of vector–matrix operations followed by a nonlinear activation function at each neuron. The process that input *x* is passed through multiple layers to produce an output prediction $a^{(L)}(x)$ is called forward propagation, also called the forward pass (Zell 1994; Svozil et al. 1997).

### Training a neural network

Training a neural network adjusts its parameters, namely the weights and biases, to minimize the difference between the network’s predictions and the actual data. From a data-fitting perspective, this process includes three main stages. First, the forward pass propagates inputs through the neural network from the input layer to the output layer, resulting in predictions. Next, the network’s outputs are evaluated using a predefined loss function to quantify how well they match the actual data. Finally, during the backward pass, also called backpropagation, the network parameters are iteratively adjusted to minimize the loss.

Mathematically, consider a dataset containing *N* training points $\{ x^{(i)}\}_{i=1}^N\subset \mathbb {R}^{n_1}$, each with corresponding desired outputs $\{ y(x^{(i)})\}_{i=1}^N \in \mathbb {R}^{n_L}$. Forward pass involves a chain of linear transformations, also called vector–matrix operations, and activation. It then calculates the output from the final layer $\{ a^{(L)}(x^{(i)})\}_{i=1}^N$.

Next, to measure how accurately the neural network maps inputs $\{ x^{(i)}\}_{i=1}^N$ to desired outputs $\{ y(x^{(i)})\}_{i=1}^N$, a cost function is used. A common choice for regression is the mean squared error, abbreviated MSE. The MSE is

2 $$\begin{aligned} \mathcal {L}(y, a^{(L)}) = \frac{1}{N} \sum _{i=1}^N \mathcal {L}_i(y, a^{(L)}), \quad \text {where} \quad \mathcal {L}_i(y, a^{(L)}) = \Vert y(x^{(i)}) - a^{(L)}(x^{(i)})\Vert ^2. \end{aligned}$$

We gather all entries from each weight matrix $W^{(l)} = [w^{(l)}_{jk}] \in \mathbb {R}^{n_l \times n_{l-1}}$, and bias vector $b^{(l)} = [b^{(l)}_{j}] \in \mathbb {R}^{n_l}$, for l=2,3,⋯,L, into a single parameter vector

$$ p = \bigl (w_{jk}^{(l)},\, b_{j}^{(l)}\bigr )_{j,k=1,\dots ,n_l;\; l=2,\dots ,L} \in \mathbb {R}^s, $$

where *s* is the total number of parameters in the entire network. For a fixed training point $x^{(i)}$ with desired output $y(x^{(i)})$, i=1,⋯,N, the cost function term $\mathcal {L}_i(y, a^{(L)})$ in (2) becomes $\mathcal {L}_i(p)$, highlighting its dependence on the parameter vector.

Minimizing the cost function $\mathcal {L}(p): \mathbb {R}^s \rightarrow \mathbb {R}$ involves iterative adjustments of *p*. For a small parameter update Δp, we approximate using a Taylor series expansion that reads

3 $$\begin{aligned} \mathcal {L}(p+\Delta p) \approx \mathcal {L}(p) + \nabla \mathcal {L}(p)^T\,\Delta p, \end{aligned}$$

where

4 $$\begin{aligned} \nabla \mathcal {L}(p) = \left( \frac{\partial \mathcal {L}}{\partial p_r}\right) _{r=1,...,s} \end{aligned}$$

is the gradient of the cost function with respect to the network parameters *p*. By the Cauchy-Schwarz inequality, to effectively reduce the cost, updates typically move in the opposite direction of the gradient $- \nabla \mathcal {L}(p)$. This leads to the gradient descent update rule

5 $$\begin{aligned} p \quad \rightarrow \quad p \; - \eta \nabla \mathcal {L}(p)^T, \end{aligned}$$

where the small stepsize η is referred to as the learning rate and $\nabla \mathcal {L}(p) = \frac{1}{N} \sum _{i=1}^N \nabla \mathcal {L}_i(p)$. Computing the gradient $\nabla \mathcal {L}(p)$ can be expensive for large datasets. Efficient optimization variants such as stochastic gradient descent (Amari 1993), mini-batch gradient descent (Khirirat et al. 2017), and adaptive methods like Adam (Kingma and Ba 2014; Kochenderfer and Wheeler 2019) are therefore widely used.

We randomly choose an initial guess for *p* and iterate via (5) until either a convergence threshold is satisfied or we run out of computational budget (Higham and Higham 2019). For instance, a necessary condition (Nocedal and Wright 1999) for a parameter vector $p^{*}$ to be a local minimizer of the loss function $\mathcal {L}(p)$ is that

6 $$\begin{aligned} \nabla \mathcal {L}(p^{*}) = \left( \frac{\partial \mathcal {L}}{\partial w^{(l)^{*}}_{jk}},\,\frac{\partial \mathcal {L}}{\partial b^{(l)^{*}}_j}\right) _{j,k=1,\dots ,n_l;\; l=2,\dots ,L} =0. \end{aligned}$$

The backward pass computes $\nabla \mathcal {L}(p)$ by reverse-mode automatic differentiation. Starting from the output-layer error

7 $$\begin{aligned} \delta ^{(L)} = \frac{\partial \mathcal {L}}{\partial z^{(L)}} = \frac{\partial \mathcal {L}}{\partial a^{(L)}} \,.\, \sigma '(z^{(L)}), \end{aligned}$$

the chain rule propagates the error backward through the layers, and a single backward sweep returns the gradients $\partial \mathcal {L} / \partial w^{(l)}_{jk}$ and $\partial \mathcal {L} / \partial b^{(l)}_{j}$ for every weight and bias for l=2,⋯,L. The detailed recursion is standard (Goodfellow et al. 2016; LeCun et al. 2012).

### Universal differential equations

In a UDE, the system dynamics are described by

8 $$\begin{aligned} \frac{d\hat{y}}{dt}=g(\hat{y},t,q, u(\hat{y},t,p)), \end{aligned}$$

where *g* is a function of the state variables $\hat{y} \in \mathbb {R}^n$, time *t*, parameter set *q* representing known parameters in the physical model, and $u(\hat{y},t,p)$ is defined by a neural network with parameters *p*. One may write $u(\hat{y},t,p)=a^{(L)}(\hat{y},t,p)$ with output dimension $n_L$. Embedding a universal approximator *u* into function *g* allows us to learn a component of the model from data while preserving the known physical laws that govern the system.

Forward pass involves solving the UDE in (8) for given parameters *p* to obtain $\hat{y}(t,p)$. Suppose there are *N* time points $\{t^{(i)}\}_{i=1}^N$ with observed data $\{y(t^{(i)})\}_{i=1}^N$. The solutions of the UDE model (8) are $\hat{y}(t^{(i)},p)$. A mean squared error cost is then defined as

9 $$\begin{aligned} \mathcal {L}(p) = \frac{1}{N} \sum _{i=1}^N \Vert y(t^{(i)}) - \hat{y}(t^{(i)},p)\Vert ^2 \end{aligned}$$

which measures the discrepancy between the observed data and the model’s predictions.

The backward pass for the UDE applies the same chain rule as in Sect. 2.2, with the extra step of differentiating $\hat{y}(t, p)$ through the ODE solver. This solver sensitivity $\partial \hat{y} / \partial u$ enters the output-layer error,

10 $$\begin{aligned} \delta ^{(L)} = \frac{\partial \mathcal {L}}{\partial z^{(L)}} = \frac{\partial \mathcal {L}}{\partial \hat{y}} \,.\, \frac{\partial \hat{y}}{\partial u} \,.\, \sigma '(z^{(L)}), \end{aligned}$$

which is then propagated backward through the network layers as in Eq. (7). Forward evaluation of the UDE, cost evaluation, and backward propagation iterate until the network meets a convergence threshold or exhausts a computation budget. This hybrid approach allows the learned component *u* to fit the training data while respecting known dynamical structure.

The exposition above frames UDE training as data fitting against observed trajectories. In the optimal control setting of Sect. 3, the observed-data targets are replaced by the cost functional itself, with the running cost playing the role of pointwise loss and the terminal payoff the role of a terminal target. The forward pass, the backward chain rule, and the parameter update of Eq. (5) carry over unchanged.

## Optimal control problem solved by deep neural network

In this section, we recast the optimal control problem as a UDE training problem. The forward pass solves the state equation, and the backward pass solves the adjoint equation by reverse-mode automatic differentiation. Minimising the loss function defined by the objective functional thereby plays the role of a forward-backward sweep, but with the adjoint obtained by backpropagation rather than backward integration.

This reformulation combines the descriptive power of differential equations for the state dynamics with the adaptability of neural networks for the control. In the saturated-control regime studied in Sect. 4, it avoids the projection-induced iterate cycling that destabilises the classical sweep, since the box constraint is enforced by a smooth parameterisation rather than by a projection step.

### Formulating the optimal control problem in the UDE framework

In the optimal control problem with *n* state variables and *m* control variables, we typically define the objective as

11 $$\begin{aligned} \begin{array}{rl} \underset{u}{\min }\ \, \mathcal {L}(u) & = \underset{u}{\min }\ \, \displaystyle \int _{t_0}^{t_1} f(t, y(t), u(t))\, dt, \\ \text {subject to} & y'(t) = g(t, y(t), u(t)), \quad y(t_0) = y_0, \end{array} \end{aligned}$$

where $y(t) = (y_1(t), \dots , y_n(t))^T$, $u(t) = (u_1(t), \dots , u_m(t))^T$, $y_0 = (y_{10}, \dots , y_{n0})^T$, and $g(t, y, u) = (g^1(t, y, u), \dots , g^n(t, y, u))^T$. The functions *f* and $g^i$ are assumed to be of class $C^1$ in all variables. For a problem that includes a terminal payoff function ϕ, the cost takes the Bolza form

12 $$\begin{aligned} \underset{u}{\min }\ \, \mathcal {L}(u) \;=\; \underset{u}{\min }\ \, \int _{t_0}^{t_1} \tilde{f}(t, y(t), u(t))\, dt \;+\; \phi (y(t_1)). \end{aligned}$$

Equation (12) reduces to the running-cost form (11) by absorbing the terminal payoff into the integrand,

$$ f(t, y(t), u(t)) \;=\; \tilde{f}(t, y(t), u(t)) \;+\; \delta (t - t_1)\, \phi (y(t)), $$

where δ denotes the Dirac delta function.

Next, we represent the control function *u*(*t*, *y*(*t*)) as a neural network. Given an input layer with one neuron per control, $a^{(1)} = (t, \dots , t)^T \in \mathbb {R}^m$, the output at layer *l* is defined as in (1) for l=2,⋯,L. The control is then taken to be the network output,

13 $$\begin{aligned} u(t, y(t)) = \text {NN}(t, p), \end{aligned}$$

where *p* collects all the weight matrices $W^{(l)}$ and bias vectors $b^{(l)}$ across the network, with $p \in \mathbb {R}^s$. Then the optimal control problem in (11) is represented in the UDE formulation with the cost function defined by the objective functional as

14 $$\begin{aligned} \begin{array}{l} \underset{\text {NN}}{\min } \, \mathcal {L}(\text {NN}) = \underset{\text {NN}}{\min } \, \displaystyle \int _{t_0}^{t_1} f(t, y(t), \text {NN}(t, p))\, dt\\ y'(t) = g(t, y(t), \text {NN}(t, p)), \quad y(t_0) = y_0 \end{array} \end{aligned}$$

or

15 $$\begin{aligned} \begin{array}{l} \underset{p}{\min }\ \, \mathcal {L}(p) = \underset{p}{\min }\ \, \displaystyle \int _{t_0}^{t_1} f(t, y(t), p)\, dt\\ y'(t) = g(t, y(t), p), \quad y(t_0) = y_0. \end{array} \end{aligned}$$

This reformulation uses the neural network as the control mechanism. Training optimises the network parameters *p* so as to minimise the cost $\mathcal {L}(p)$ over $[t_0, t_1]$. We solve the state equation in (15) forward in time to obtain the state trajectory *y*(*t*), and then compute the gradient $\nabla \mathcal {L}(p)$ via reverse-mode automatic differentiation to update *p*. Theoretically, the training finishes when we find a stationary point $p^*$ of $\mathcal {L}$, i.e., $\nabla \mathcal {L}(p^*) = 0$. The resulting controller $u^*(t) = \text {NN}(t, p^*)$ and state $y^*(t)$ will be shown to satisfy the PMP necessary conditions in the next subsections.

### Gradient descent

To choose an update Δp for the network parameters that decreases the cost at each iterate, we need to compute the gradient $\nabla \mathcal {L}(p) \in \mathbb {R}^s$ as in (4). We will prove that a parameter set $p^*$ satisfying $\nabla \mathcal {L}(p^*)=0$ yields an optimal control $u^*(t) = \text {NN}(t, p^*)$ and an associated state $y^*(t) = y(t, p^*)$ satisfying PMP.

With *n* states in the system, we introduce *n* corresponding adjoint variables, one for each state. We collect these into the piecewise differentiable vector-valued function $\lambda (t) = [\lambda _1(t), \dots , \lambda _n(t)]^T$, where each $\lambda _i(t)$ corresponds to the state variable $y_i(t)$. For each network parameter $p_r$, r=1,⋯,s, the *r*-th component of the gradient $\nabla \mathcal {L}(p)$ with $\mathcal {L}$ defined in (15) is

16 $$\begin{aligned} \dfrac{\partial \mathcal {L}}{\partial p_r}= &  \dfrac{\partial }{\partial p_r} \int _{t_0}^{t_1}f(t,y(t, p),p)dt \nonumber \\= &  \dfrac{\partial }{\partial p_r} \left( \int _{t_0}^{t_1} \left[ f(t,y(t, p),p) +\dfrac{d}{dt} (\lambda (t)^T\, y(t, p)) \right] dt -\left[ \lambda (t)^T\, y(t, p)\right] |_{t_0}^{t_1} \right) \nonumber \\= &  \int _{t_0}^{t_1} \left[ \dfrac{\partial }{\partial p_r} f(t,y(t, p),p) + \dfrac{\partial }{\partial p_r} \left( \dfrac{d \lambda (t)}{dt}^T\, y(t, p) + \lambda (t)^T\, \dfrac{d y(t, p)}{dt} \right) \right] \nonumber \\ &  \quad dt - \dfrac{\partial }{\partial p_r} \left[ \lambda (t)^T\, y(t, p)\right] |_{t_0}^{t_1} \nonumber \\= &  \int _{t_0}^{t_1} \left[ \nabla f_y^T\, \dfrac{\partial y(t, p)}{\partial p_r} + \dfrac{\partial f}{\partial p_r} + \dfrac{d \lambda (t)}{dt}^T\, \dfrac{\partial y(t, p)}{\partial p_r} + \lambda (t)^T\, \dfrac{d g}{d p_r} \right] dt \nonumber \\ &  - \lambda (t_1)^T\, \dfrac{\partial y(t_1, p)}{\partial p_r} +\lambda (t_0)^T\, \dfrac{\partial y(t_0, p)}{\partial p_r}, \end{aligned}$$

where $\nabla f_y = (f_{y_1}, \dots , f_{y_n})^T$, *y*(*t*, *p*) is solved from the state equation in (15), and the superscript $\phantom {0}^T$ denotes transpose, so that $a^T b$ is the dot product of two column vectors $a, b \in \mathbb {R}^n$. Writing *g* in components as $g(t, y, p) = (g^1, \dots , g^n)^T$, the last term in the integrand in (16) expands as

17 $$\begin{aligned} \lambda (t)^T\, \dfrac{d g }{d p_r}= &  \lambda (t)^T\, \begin{pmatrix} (\nabla g^1_y)^T\, \dfrac{\partial y(t)}{\partial p_r} + \nabla g^1_{p_r} \\ \vdots \\ (\nabla g^n_y)^T\, \dfrac{\partial y(t)}{\partial p_r} + \nabla g^n_{p_r} \end{pmatrix} \nonumber \\= &  \lambda (t)^T\, \left( \nabla (g^k_y)^T\, \dfrac{\partial y(t)}{\partial p_r} \right) ^T_{k=1,\dots , n} + \lambda (t)^T\, \left( \nabla g^k_{p_r} \right) ^T_{k=1,\dots , n} \nonumber \\= &  \displaystyle \sum \limits _{k=1}^n \lambda _k(t)\, \nabla (g^k_y)^T\, \dfrac{\partial y(t)}{\partial p_r} + \lambda (t)^T\, \left( \nabla g^k_{p_r} \right) ^T_{k=1,\dots , n} \nonumber \\= &  \sum \limits _{k=1}^n \lambda _k(t)\, \left( \sum \limits _{j=1}^n \dfrac{\partial g^k}{\partial y_j} \dfrac{\partial y_j(t)}{\partial p_r} \right) + \lambda (t)^T\, \left( \nabla g^k_{p_r} \right) ^T_{k=1,\dots , n} \nonumber \\= &  \displaystyle \sum \limits _{j=1}^n \left( \sum \limits _{k=1}^n \lambda _k(t)\,\dfrac{\partial g^k}{\partial y_j} \right) \dfrac{\partial y_j(t)}{\partial p_r} + \lambda (t)^T\, \left( \nabla g^k_{p_r} \right) ^T_{k=1,\dots , n} \nonumber \\= &  \left( \lambda (t)^T\, \dfrac{\partial g}{\partial y_j} \right) _{j=1,\dots ,n} \dfrac{\partial y(t)}{\partial p_r} + \lambda (t)^T\, \left( \nabla g^k_{p_r} \right) ^T_{k=1,\dots , n}. \end{aligned}$$

Recalling $y(t_0,p) \equiv y(t_0)$, we have $\frac{\partial y(t_0, p)}{\partial p_r}=0$. Further, denoting $\left( \nabla g^k_{p_r} \right) ^T_{k=1,\dots , n} = \frac{\partial g}{\partial p_r}$, Eq. (16) becomes

18 $$\begin{aligned} \begin{array}{rl} \dfrac{\partial \mathcal {L}}{\partial p_r} =& \displaystyle \int _{t_0}^{t_1} \left[ \nabla f_y + \dfrac{d \lambda (t)}{dt} + \left( \lambda (t)^T\, \dfrac{\partial g}{\partial y_j} \right) _{j=1,\dots ,n}^T \right] ^T\, \dfrac{\partial y(t)}{\partial p_r} dt \\ + & \displaystyle \int _{t_0}^{t_1} \left[ \dfrac{\partial f}{\partial p_r} + \lambda (t)^T\, \left( \nabla g^k_{p_r} \right) ^T_{k=1,\dots , n} \right] dt - \lambda (t_1)^T\, \dfrac{\partial y(t_1, p)}{\partial p_r}. \end{array} \end{aligned}$$

### The necessary conditions

Suppose the network parameter set $p^*$ satisfies $\nabla \mathcal {L}(p^*) = 0$, and let $u^*(t) = \text {NN}(t, p^*)$ and $y^*(t) = y(t, p^*)$ denote the corresponding controller and state. We then choose the adjoint function λ(t) to satisfy

19 $$\begin{aligned} \dfrac{d\lambda _i(t)}{dt} =&- \dfrac{\partial }{\partial y_i} \left[ f(t, y^*(t), p^*) + \lambda (t)^T\, g(t, y^*(t), p^*) \right] , \; \text {for}\; i = 1, \dots , n \quad \nonumber \\&\text {(adjoint equation)}, \end{aligned}$$

and the boundary condition

20 $$\begin{aligned} \lambda (t_1) = 0 \quad \text {(transversality condition)}. \end{aligned}$$

Now (18) reduces to

21 $$\begin{aligned} 0= \int _{t_0}^{t_1} \left[ \dfrac{\partial f}{\partial p_r} + \lambda (t)^T\, \left( \nabla g^k_{p_r} \right) ^T_{k=1,\dots , n} \right] \Bigg |_{y^*, p^*} dt. \end{aligned}$$

Denoting $\left( \nabla g^k_{p_r} \right) ^T_{k=1,\dots , n}$ as $\frac{\partial g}{\partial p_r}$, Eq. (21) implies the optimality condition

22 $$\begin{aligned} \dfrac{\partial }{\partial p_r}f(t, y^*(t), p^*) + \lambda (t)^T\, \dfrac{\partial }{\partial p_r}g(t, y^*(t), p^*) = 0 \quad \text {for all} \quad t_0 \le t \le t_1. \end{aligned}$$

The above derivation yields the following result.

#### Theorem 1

Suppose the network parameter set $p^*$ satisfies $\nabla \mathcal {L}(p^*) = 0$, and let $u^*(t) = \textrm{NN}(t, p^*)$ and $y^*(t) = y(t, p^*)$ denote the corresponding controller and state for problem (15). Then there exists a piecewise differentiable adjoint variable λ(t) such that, at each $t \in [t_0, t_1]$, $u^*(t)$ is a critical point of the map

$$ u \;\longmapsto \; H\bigl (t, y^*(t), u, \lambda (t)\bigr ), $$

where the Hamiltonian is defined by

$$\begin{aligned} H\bigl (t, y(t), u(t), \lambda (t)\bigr ) = f\bigl (t, y(t), u(t)\bigr ) + \lambda (t)^T\, g\bigl (t, y(t), u(t)\bigr ), \end{aligned}$$

and λ(t) is governed by the adjoint Eq. (19) together with the transversality condition (20).

### Discretisation-gap bound

Theorem 1 is a continuous-time statement. It says that if the network parameters $p^{*}$ make the *continuous* loss-gradient vanish, then the controller satisfies the PMP necessary conditions. In practice, training minimises a *discretised* loss obtained by approximating the time integral with a trapezoidal-rule sum on a uniform time grid and replacing the exact state trajectory with an ODE-solver output computed at prescribed tolerances. The trapezoidal rule plays a single role throughout the training pipeline. It turns the continuous-time cost integral of Eq. (15) into a differentiable scalar that the gradient-descent optimiser can minimise. We now quantify the gap between the two.

Fix a uniform time grid $t_k = t_0 + k h$ for k=0,…,M with $h = (t_1 - t_0) / M$, and let $w_k$ denote the trapezoidal-rule weights, with $w_0 = w_M = 1/2$ and $w_k = 1$ otherwise. Let $\tilde{y}(t; p)$ denote the state trajectory produced by an adaptive ODE solver with relative tolerance $\textrm{rtol}$ and absolute tolerance $\textrm{atol}$, and let $y_{*}(t; p)$ denote the exact state trajectory. In our implementation, the forward state is integrated with an adaptive fourth/fifth-order Runge–Kutta Dormand-Prince scheme (Dormand and Prince 1980). The backward adjoint is integrated with a backward differentiation formula suited to stiff problems (Hairer and Wanner 1996). The discrete loss used by the optimiser is

$$ \mathcal {L}_h(p) \;=\; h \sum _{k=0}^{M} w_k\, f\bigl (t_k, \tilde{y}(t_k; p), p\bigr ) \;+\; \phi \bigl (\tilde{y}(t_1; p)\bigr ), $$

where ϕ is the terminal payoff of Eq. (12), written here as an explicit summand because the trapezoidal sum on a finite grid cannot reproduce the Dirac-delta encoding. The continuous loss of Eq. (15) is denoted $\mathcal {L}_{\text {cts}}(p)$.

#### Proposition 2

(Discretisation gap) Assume that *f*, *g*, and ϕ are of class $C^2$ on a bounded set containing the trajectory, that the state ODE in (15) is asymptotically stable on $[t_0, t_1]$ for every parameter vector *p* in a bounded neighbourhood, and that the adaptive ODE solver satisfies the standard global-error bound

23 $$\begin{aligned} \sup _{t \in [t_0, t_1]} \bigl \Vert \tilde{y}(t; p) - y_{*}(t; p) \bigr \Vert \;\le \; K_y\bigl (\, \textrm{rtol} + \textrm{atol}\, \bigr ), \end{aligned}$$

where $K_y > 0$ is the solver’s global-error constant for the forward state, with an analogous bound

$$ \sup _{t \in [t_0, t_1]} \bigl \Vert \tilde{\lambda }(t; p) - \lambda _{*}(t; p) \bigr \Vert \;\le \; K_\lambda \bigl (\, \textrm{rtol} + \textrm{atol}\, \bigr ) $$

holding for the adjoint trajectory with analogous constant $K_\lambda > 0$. Then there exist constants $C_1, C_2, C_3 > 0$, depending on the model and on $\sup \Vert y_{*}\Vert$, $\sup \Vert \lambda _{*}\Vert$ and their derivatives but not on *p*, such that

24 $$\begin{aligned} \Bigl \Vert \nabla _p \mathcal {L}_{\textrm{cts}}(p) - \nabla _p \mathcal {L}_h(p) \Bigr \Vert \;\le \; C_1\, \textrm{rtol} + C_2\, \textrm{atol} + C_3\, h^2, \qquad \forall p \in \mathbb {R}^s. \end{aligned}$$

In particular, if $p^{*}$ is a stationary point of the discrete loss with $\nabla _p \mathcal {L}_h(p^{*}) = 0$, then

25 $$\begin{aligned} \bigl \Vert \nabla _p \mathcal {L}_{\textrm{cts}}(p^{*}) \bigr \Vert \;\le \; C_1\, \textrm{rtol} + C_2\, \textrm{atol} + C_3\, h^2. \end{aligned}$$

#### Proof

Using the adjoint identity established in Eq. (18), the per-coordinate continuous gradient is

$$ \frac{\partial \mathcal {L}_{\text {cts}}}{\partial p_r}(p) \;=\; \int _{t_0}^{t_1} \Phi _r\bigl (t, y_{*}(t; p), \lambda _{*}(t; p), p\bigr )\, dt, \qquad \Phi _r:= \frac{\partial f}{\partial p_r} + \lambda ^{\top } \frac{\partial g}{\partial p_r}. $$

The discrete gradient produced by reverse-mode automatic differentiation through the trapezoidal-quadrature loss is

$$ \frac{\partial \mathcal {L}_h}{\partial p_r}(p) \;=\; h \sum _{k=0}^{M} w_k\, \Phi _r\bigl (t_k, \tilde{y}(t_k; p), \tilde{\lambda }(t_k; p), p\bigr ). $$

We split the difference into a quadrature error and an ODE-solver error. The quadrature error is evaluated along the exact trajectory. The ODE-solver error is evaluated with the quadrature held fixed. The decomposition reads

$$\begin{aligned} \frac{\partial \mathcal {L}_{\text {cts}}}{\partial p_r} - \frac{\partial \mathcal {L}_h}{\partial p_r}&= \underbrace{\Bigl [\, \int _{t_0}^{t_1}\!\!\Phi _r(t, y_{*}, \lambda _{*}, p)\, dt \;-\; h \sum _{k} w_k\, \Phi _r(t_k, y_{*}, \lambda _{*}, p) \Bigr ]}_{=:\, E_h^{(r)}\ \text {[quadrature error]}} \\&\quad + \underbrace{h \sum _{k} w_k\, \bigl [\Phi _r(t_k, y_{*}, \lambda _{*}, p) \;-\; \Phi _r(t_k, \tilde{y}, \tilde{\lambda }, p)\bigr ]}_{=:\, E_{\text {ode}}^{(r)}\ \text {[ODE-solver error]}}. \end{aligned}$$

**Bound on** $E_h^{(r)}$ **.** The standing $C^2$ assumption makes the integrand $t \mapsto \Phi _r(t, y_{*}(t), \lambda _{*}(t), p)$ a member of $C^2([t_0, t_1])$. The standard error bound for the composite trapezoidal rule (Freund and Hoppe 2007) yields

26 $$\begin{aligned} \bigl | E_h^{(r)} \bigr | \;\le \; \tfrac{(t_1 - t_0)}{12}\, h^2\, \sup _{t \in [t_0, t_1]} \bigl | \tfrac{d^2}{dt^2} \Phi _r(t, y_{*}(t), \lambda _{*}(t), p) \bigr | \;=:\; C_3^{(r)}\, h^2, \end{aligned}$$

with $C_3^{(r)}$ uniform in *p* on a bounded neighbourhood by the regularity assumption.

**Bound on** $E_{\text {ode}}^{(r)}$ **.** Apply the mean-value form of Taylor’s theorem to $\Phi _r$ in its (y,λ) arguments,

$$ \Phi _r(t_k, \tilde{y}, \tilde{\lambda }, p) - \Phi _r(t_k, y_{*}, \lambda _{*}, p) = \frac{\partial \Phi _r}{\partial y}\bigl (\tilde{y} - y_{*}\bigr ) + \frac{\partial \Phi _r}{\partial \lambda }\bigl (\tilde{\lambda } - \lambda _{*}\bigr ) + R_k, $$

with the remainder $R_k$ controlled by the second derivatives of $\Phi _r$ and the squares of the trajectory errors, $|R_k| \le M_2 (\Vert \tilde{y} - y_{*}\Vert ^2 + \Vert \tilde{\lambda } - \lambda _{*}\Vert ^2)$. Combining with the ODE-solver bound (23) and the analogous bound on the adjoint, we obtain

$$ \bigl | \Phi _r(t_k, \tilde{y}, \tilde{\lambda }, p) - \Phi _r(t_k, y_{*}, \lambda _{*}, p) \bigr | \;\le \; M_1\bigl (K_y + K_\lambda \bigr )\bigl (\textrm{rtol} + \textrm{atol}\bigr ) + O\bigl ((\textrm{rtol} + \textrm{atol})^2\bigr ), $$

where $M_1:= \sup \bigl (\Vert \partial \Phi _r/\partial y\Vert , \Vert \partial \Phi _r / \partial \lambda \Vert \bigr )$. Multiplying by $h \sum _k w_k = (t_1 - t_0)$ and dropping the $O\bigl ((\textrm{rtol} + \textrm{atol})^2\bigr )$ terms gives

27 $$\begin{aligned} \bigl | E_{\text {ode}}^{(r)} \bigr | \;\le \; C_1^{(r)}\, \textrm{rtol} + C_2^{(r)}\, \textrm{atol}, \end{aligned}$$

with $C_1^{(r)} = C_2^{(r)} = M_1 (K_y + K_\lambda ) (t_1 - t_0)$, the two constants coinciding because the ODE-solver bound (23) lumps $\textrm{rtol}$ and $\textrm{atol}$ symmetrically.

**Combining.** Summing (26) and (27) over coordinates r=1,…,s and taking the Euclidean norm gives the bound (24) with $C_i = (\sum _r (C_i^{(r)})^2)^{1/2}$. Inequality (25) follows by applying (24) at $p = p^{*}$ and using $\nabla _p \mathcal {L}_h(p^{*}) = 0$. □

## An application on cancer immunotherapy

We apply the UDE framework to design a combination immunotherapy schedule for a cancer immune model with bifurcation-driven dynamics (Zhang et al. 2024). The resulting numerical stiffness in the forward state and backward adjoint equations destabilises the traditional forward-backward sweep. We replace the backward sweep with backpropagation through the UDE. Gradient descent then operates on the objective functional directly.

Cancer immunotherapy activates T cells to recognise and destroy cancer cells. Activation requires both antigen recognition and co-stimulation. Without either signal, the T cell becomes anergic. Immunotherapy enhances this cascade through CAR T-cell therapies (Harrison et al. 2021), monoclonal antibodies (Effer et al. 2023), and immune checkpoint inhibitors such as anti-PD-1 and anti-CTLA-4 (Curran et al. 2010). Combination strategies simultaneously promote co-stimulation and block co-inhibition. They may trigger cytokine release syndrome (Maude et al. 2014) or other immune-related adverse events (Marshall et al. 2018). These risks motivate mathematically guided dose optimisation (Zheng and Kim 2021; Pappalardo et al. 2019; Alfonso et al. 2020; Jenner et al. 2021; Zahid et al. 2021; Bradshaw et al. 2019; Xiong et al. 2021).

### Combination cancer immunotherapy model

An established immune-cancer model (Zheng and Kim 2021) is

28 $$\begin{aligned} \begin{array}{ll} \dfrac{dC}{dt} & = F(C)\,C-\kappa CE, \\ \dfrac{dA}{dt} & = r_A C-\delta _A A, \\ \dfrac{dI}{dt} & = r_I CE-\delta _I I, \\ \dfrac{dE}{dt} & = -r_E\left( E-E^{*} \right) + u_1(t)\beta AIES -u_2(t)\gamma ES,\\ \dfrac{dS}{dt} & = -r_S\left( S-S^{*} \right) - u_1(t)\beta AIES +u_2(t)\gamma ES. \end{array} \end{aligned}$$

The state variables are the cancer cell concentration *C*(*t*), the tumor-specific antigen *A*(*t*), the inflammatory signal *I*(*t*), and the effector and non-effector T-cell populations *E*(*t*), *S*(*t*) within the tumor microenvironment. Cancer cells grow logistically at rate

$$ F(C) \;=\; \min \bigl \{\, r_{\max },\; r_C\bigl (1 - C/C^{*}\bigr ) \,\bigr \}. $$

They are killed by effector T cells through the mass action term κCE. The antigen and inflammatory signal are produced at rates $r_A C$ and $r_I CE$, and degrade at rates $\delta _A$ and $\delta _I$. The two T-cell populations relax to their steady states $E^{*}, S^{*}$ at rates $r_E, r_S$. The term βAIES converts *S* into *E* when antigen and inflammation are present. The term γES reverts *E* back to *S*.

The parameters β and γ are the two pharmacological targets of combination immunotherapy. The rate β controls *co-stimulation*. A larger β moves more cells from *S* into *E* and raises the tumor killing capacity. The rate γ controls *co-suppression*. A larger γ reverts more *E* back into *S* and spares the tumor. Anti-CTLA-4 antibodies amplify β via the multiplier $u_1(t)$. Anti-PD-1 antibodies suppress γ via the multiplier $u_2(t)$. The next subsection defines $u_1, u_2$ in terms of bounded receptor occupancy controls $v_1(t), v_2(t) \in [0, 1]$.

### Optimal control formulation

Our goal is to design treatment schedules that satisfy three clinical objectives. The schedule must reduce tumor burden both over and at the end of the treatment horizon. It must leave a competent effector T-cell population at the end of treatment so that residual disease can be controlled after dosing stops. It must also limit cumulative drug exposure. We encode these three goals through dimensionless *blockade fractions* $v_1(t), v_2(t) \in [0, 1]$ rather than letting the state equation multipliers $u_1, u_2$ be unbounded free controls. The fraction $v_1$ is the anti-CTLA-4 receptor occupancy and acts as a co-stimulation amplifier on the rate β. The fraction $v_2$ is the anti-PD-1 receptor occupancy and acts as a co-suppression damper on the rate γ. The state equation multipliers in (28) are then

29 $$\begin{aligned} u_1(t) \;=\; 1 + \alpha _1\, v_1(t), \qquad u_2(t) \;=\; 1 - \alpha _2\, v_2(t), \end{aligned}$$

with drug class efficacy ceilings $\alpha _1 = 2$ for anti-CTLA-4 and $\alpha _2 = 0.8$ for anti-PD-1. The amplification ceiling $\alpha _1$ has no hard mathematical upper bound. The damping ceiling $\alpha _2$ must satisfy $\alpha _2 \le 1$, so that $u_2 \ge 0$ and the E→S conversion rate stays non-negative. Within these constraints, both values are chosen as moderate, illustrative ceilings. Setting $v_1 = 0$ recovers the untreated co-stimulation rate $u_1 = 1$, and $v_1 = 1$ amplifies it to $u_1 = 3$. Setting $v_2 = 0$ recovers $u_2 = 1$, and $v_2 = 1$ reduces $u_2$ to 0.2. This separation of drug class properties from dosing schedule is consistent with standard PK/PD modelling.

The optimal control problem is to minimise the objective functional

30 $$\begin{aligned} \min _{(v_1, v_2)\in [0,1]^2}\; J(v_1, v_2) \;=\; \int _0^{t_f} \!\! \Bigl [\, \alpha _C\, C(t) + \tfrac{\alpha _T}{2}\bigl (v_1^2(t) + v_2^2(t)\bigr ) \Bigr ]\, dt \;+\; \psi _C\, C(t_f) \;-\; \psi _E\, E(t_f), \nonumber \\ \end{aligned}$$

subject to the state Eq. (28) with the controls in (29). We take the final time $t_f = 100$ days. The baseline initial conditions (ICs) are listed in Table 1. Each component of *J* corresponds to one of the three clinical goals stated above. The running cost $\alpha _C C(t)$ penalises tumor burden integrated over the entire treatment horizon. Fast eradication is therefore rewarded relative to slow clearance. The toxicity term $\tfrac{\alpha _T}{2}(v_1^2 + v_2^2)$ follows the standard quadratic dose-toxicity convention adopted across the cancer optimal control literature (Lenhart and Workman 2007; Nanda et al. 2007). It is now expressed in the blockade fractions $v_i$ and captures the empirically-observed superlinear growth of immune-related adverse events with cumulative receptor occupancy (Naidoo et al. 2015). The terminal pair $\psi _C C(t_f) - \psi _E E(t_f)$ encodes the end of treatment target. The term $\psi _C C(t_f)$ penalises any residual tumor. The negative-sign reward $-\psi _E E(t_f)$ explicitly encourages a competent effector population at $t_f$ rather than penalising tumor burden alone.

The four cost weights are $(\alpha _C, \alpha _T, \psi _C, \psi _E) = (10^{-3}, 1, 10^{-2}, 1)$. They are chosen by scale-balancing so that each integrand and terminal term contributes *O*(1) to *J* at typical state magnitudes such as $C \sim 10^3$, $v_i \sim 1$, and $E \sim 10^0$. Robustness to this choice is examined in Sect. 4.7.

**Table 1:** Initial-condition grid for the FBS-vs-UDE comparison

Label	*C*(0)	A(0)=I(0)	*E*(0)	*S*(0)
Baseline (Zheng and Kim 2021)	1000	1	5	5
Low tumor	100	1	5	5
High tumor	5000	5	1	5
High effector	1000	1	20	5

### Forward-backward sweep implementation and failure modes

We first solve (30) with the classical forward-backward sweep, abbreviated FBS, then with UDE backpropagation as described in Sect. 4.4. The benchmark is the empirical content of this section.

Write the state Eq. (28) compactly as $\dot{y} = g(y, v)$ with $y = (C, A, I, E, S)^{\top }$ and $v = (v_1, v_2)^{\top }$. The Pontryagin necessary conditions yield the Hamiltonian

$$ H(y, v, \lambda ) \;=\; \alpha _C\, C + \tfrac{\alpha _T}{2}\,(v_1^2 + v_2^2) + \lambda ^{\top } g(y, v). $$

The adjoint equations are $\dot{\lambda }_i = -\partial H / \partial y_i$ with transversality $\lambda (t_f) = (\psi _C, 0, 0, -\psi _E, 0)^{\top }$. The pointwise optimality conditions, obtained from $\partial H / \partial v_i = 0$ followed by elementwise projection onto [0, 1], are

31 $$\begin{aligned} \begin{aligned} v_1^{*}(t)&= \max \!\Bigl \{0,\, \min \!\Bigl \{1,\, \tfrac{(\lambda _5 - \lambda _4)\, \alpha _1\, \beta \, A I E S}{\alpha _T}\Bigr \}\Bigr \}, \\ v_2^{*}(t)&= \max \!\Bigl \{0,\, \min \!\Bigl \{1,\, \tfrac{(\lambda _5 - \lambda _4)\, \alpha _2\, \gamma \, E S}{\alpha _T}\Bigr \}\Bigr \}. \end{aligned} \end{aligned}$$

Full derivations of the five adjoint equations and the optimality conditions are deferred to Appendix A.

The classical FBS (Lenhart and Workman 2007) iterates these conditions to a fixed point. Starting from $v_1^{(0)} \equiv v_2^{(0)} \equiv 0.5$, each iteration *k* performs four steps. The state equations are solved forward in time given $v^{(k)}(t)$. The adjoint equations are solved backward in time given the state and $v^{(k)}(t)$. The projected optimality update $v^{*}(t)$ is computed from (31). The next iterate is then formed as the convex combination

$$ v^{(k+1)} \;=\; \omega \, v^{*} + (1 - \omega )\, v^{(k)}. $$

The damping parameter $\omega \in (0, 1]$ controls how aggressively the iterate moves toward the projected target. Convergence is monitored through the relative change metric

32 $$\begin{aligned} r^{(k+1)} \;=\; \frac{\Vert v^{(k+1)} - v^{(k)}\Vert _1}{\Vert v^{(k+1)}\Vert _1}. \end{aligned}$$

The metric $r^{(k+1)}$ is the natural scale invariant measure of how much the iterate is still moving. When FBS converges to a fixed point $v^{*}$, $r^{(k+1)} \rightarrow 0$. When FBS is trapped in a 2-cycle ($v^{(k+2)} = v^{(k)}$ but $v^{(k+1)} \ne v^{(k)}$), $r^{(k+1)}$ stays bounded away from zero forever. The iteration terminates at the first *k* with $r^{(k)} < \textrm{tol} = 10^{-3}$.

We run FBS in two damping regimes. The *nominal* regime fixes ω=0.5 with no adaptive damping and an iteration cap of 30. This is the textbook setting that succeeds on most well-behaved OC problems in the literature. The *damped* regime starts with ω=0.1 with adaptive halving when oscillation is detected, down to a floor $\omega = 10^{-3}$. It uses an iteration cap of 500 and a wallclock cap of two hours per scenario. This is the production-grade fix that mirrors what practitioners use when the textbook setting fails.

In both regimes we use forward state integration with RK45 at $\textrm{rtol} = 10^{-9}$ and $\textrm{atol} = 10^{-10}$. We use backward adjoint integration with stiff implicit BDF at $\textrm{rtol} = \textrm{atol} = 10^{-11}$.

#### The nominal regime fails by saturated projection

The stationarity condition $\partial H / \partial v_i = 0$ gives the unconstrained optimum

33 $$\begin{aligned} v_i^{**}(t) \;=\; \frac{(\lambda _5 - \lambda _4)\,\alpha _i\,\rho _i\bigl (y(t)\bigr )}{\alpha _T}, \qquad \rho _1 = \beta \, A I E S, \quad \rho _2 = \gamma \, E S. \end{aligned}$$

This is the argument of the $$\max \{0, \min \{1, \cdot \}\}$$ projection in (31), so $$v_i^{*} = \max \{0, \min \{1, v_i^{**}\}\}$$. When $$|v_i^{**}|$$ exceeds 1, the projection clips $$v_i^{*}$$ to a boundary value, 0 or 1. At the Zheng-Kim parameter values used here, listed in Table 4, the clip happens almost everywhere along the trajectory. The dominant scale is $$\gamma \approx 37.4$$. With *E* and *S* in $$[O(10^{-2}), O(10^1)]$$, the factor $$\rho _2 = \gamma E S$$ reaches values around $$[5, 10^3]$$. With $$\alpha _2 = 0.8$$, $$\alpha _T = 1$$, and an order-unity adjoint difference, this drives $$|v_2^{**}|$$ to values between 10 and $$10^3$$, well outside [0, 1]. The transversality $$\lambda _5(t_f) - \lambda _4(t_f) = \psi _E = 1$$ gives one such order-unity value. The same scaling applies to $$v_1^{**} \propto \beta A I E S$$ for the high-tumor and high-effector ICs of Table 1. Across most of the time grid, $$v_i^{*}(t)$$ therefore takes only the boundary values 0 or 1, with narrow transition windows where $$v_i^{**}(t) \in (0, 1)$$. We call this pattern a *saturated control*.

Each FBS iterate is therefore a saturated control. The question is whether the iteration converges to a single such pattern. It does not. Instead, FBS alternates between two saturation patterns and never converges. To make this concrete, define the *support set* $\mathcal {S}_i^{(k)} = \{t \in [0, t_f]: v_i^{(k)}(t) = 1\}$, the part of the time grid where the *k*th iterate hits its upper boundary. When the iterate is saturated, $\mathcal {S}_i^{(k)}$ characterises $v_i^{(k)}$ up to a measure-zero transition region. The forward state and backward adjoint passes are nonlinear in *y*. A small change in $\mathcal {S}_i^{(k)}$ produces a large change in the next adjoint difference $(\lambda _5 - \lambda _4)$, which flips $\mathcal {S}_i^{(k+1)}$ at multiple time points. The following iteration flips the support back. The iteration alternates between two saturation patterns indefinitely. Textbook FBS convergence proofs (Lenhart and Workman 2007; McAsey et al. 2012) require the fixed point operator to be Lipschitz with constant strictly less than 1. The projection has a discontinuous derivative at the constraint boundary, and this contractivity fails whenever the support set is unstable across iterations. The relative change metric (32) therefore stays bounded away from zero, producing the sustained 2-cycle we characterise empirically below.

We assess FBS on a 4×4 grid of $(\gamma , \textrm{IC})$ scenarios. The four ICs are listed in Table 1. The four bifurcation-parameter values are $\gamma \in \{10, 20, 37.417, 40\}$. The value γ=37.417 lies near the saddle-node bifurcation $\textrm{SN}_2$ at γ=37.4167 (Zhang et al. 2024), where the limit-cycle period diverges and the model produces a delayed response pattern of roughly 100 days of cancer dormancy followed by rapid resurgence. The state and adjoint dynamics are stiff in this regime, so we refer to this column as the *delayed response scenario*. The remaining values $\gamma \in \{10, 20, 40\}$ span pre- and post-$\textrm{SN}_2$ regimes. Each of the 16 scenarios is one independent FBS run.

**Fig. 1:**
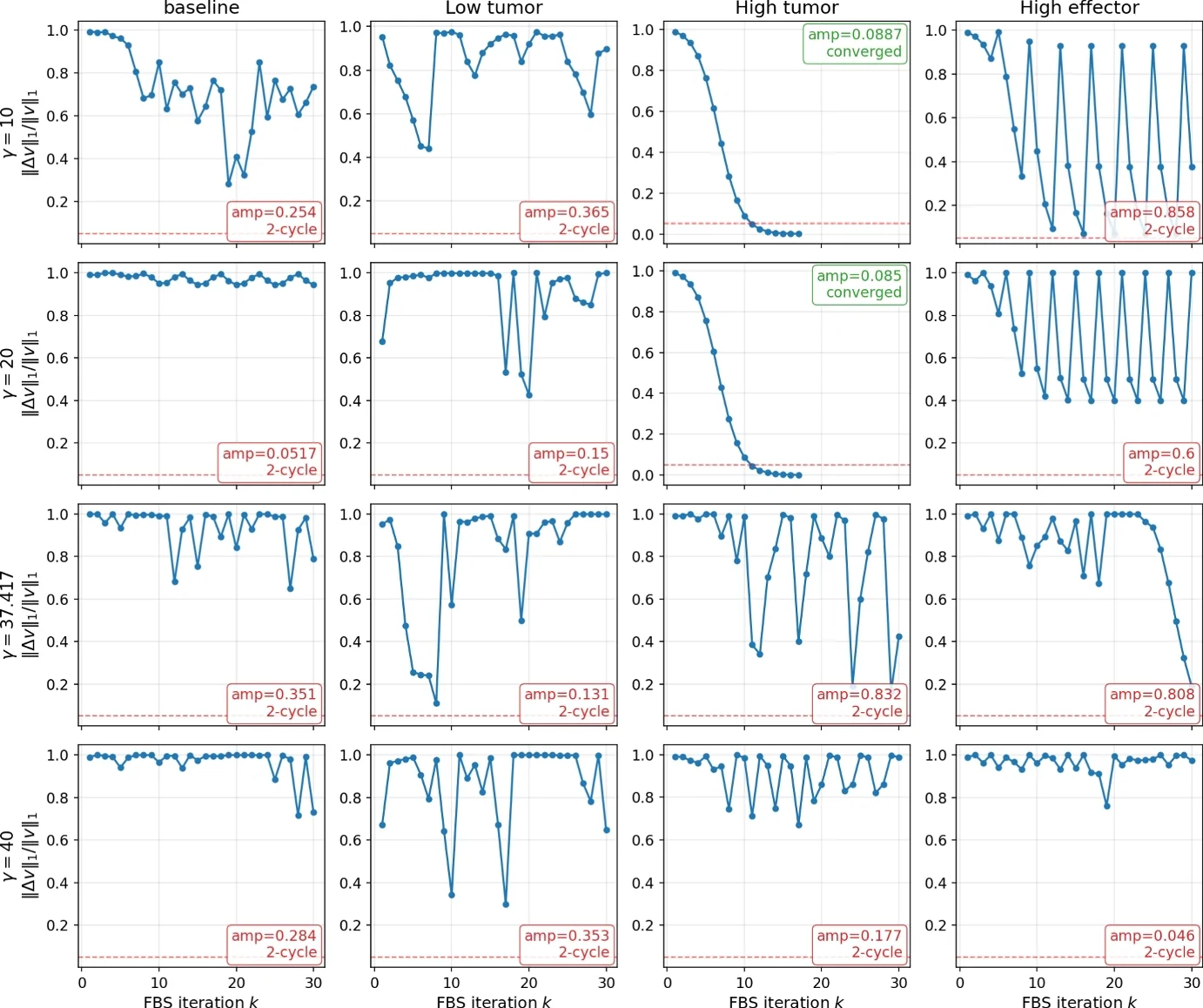
FBS fixed point relative change per iteration in the nominal regime

The primary convergence diagnostic is Fig. 1, which plots the relative change metric $r^{(k)}$ from (32) against iteration index *k* for each $(\gamma , \textrm{IC})$ scenario in the nominal regime with ω=0.5 and no adaptive damping. Rows index γ and columns index IC. By construction, $r^{(k)} \rightarrow 0$ if and only if the iterate $v^{(k)}$ approaches a fixed point. A converging scenario shows $r^{(k)}$ decaying monotonically and crossing the dashed tolerance line. A non-converging scenario shows $r^{(k)}$ pinned at or above the threshold throughout the run. Each panel carries a per-scenario verdict read directly off the FBS termination flag. Green marks convergence via $r^{(k)} < \textrm{tol}$ and red marks a saturated 2-cycle. Across the 4×4 grid, 14 of 16 scenarios fail to converge by the criterion that $r^{(k)}$ remains stuck above 0.05 throughout the last 8 iterations. The two converging scenarios are both high_tumor at low γ, namely γ=10 and γ=20. These are interior-optimum cases where the optimum does not push controls onto the boundary. Both early-stop within 17 iterations with terminal $r^{(k)} < 10^{-3}$. The remaining 14 scenarios are saturated 2-cycles where the support set $\mathcal {S}_i^{(k)}$ flips between two patterns from one iteration to the next. One panel, high_effector at γ=40, appears stationary at first glance because $r^{(k)}$ values cluster narrowly around 0.97, but is in fact a 2-cycle. Reading down the columns, the failure rate scales monotonically with γ.

**Fig. 2:**
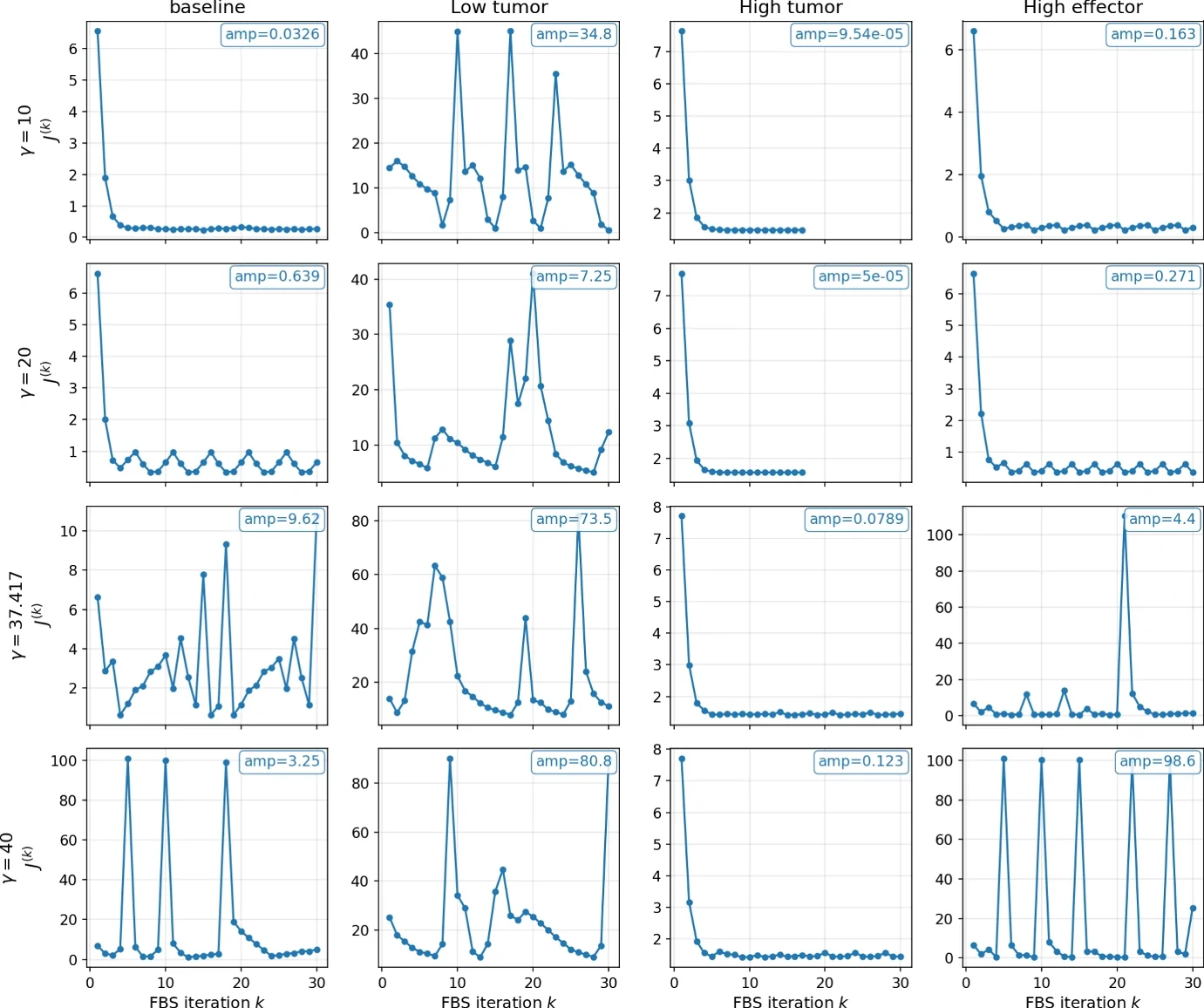
FBS objective $J^{(k)}$ across iterations in the nominal regime

Figure 2 is the consistency check on the cost. Each panel plots $J^{(k)}$ against iteration *k*. For a converging scenario, $J^{(k)}$ should decrease monotonically and plateau. For a non-converging scenario, $J^{(k)}$ typically swings between two values, mirroring the 2-cycle in the controls. This swing is visible in the low_tumor and high_effector columns at $\gamma \ge 37.417$. At the high_tumor scenarios and at γ=10, baseline IC, $J^{(k)}$ instead appears nearly flat. The cause is structural rather than convergence-related. The tumor burden integral $\int \alpha _C C(t)\,dt$ dominates *J* in those scenarios, and the two saturation patterns produce nearly identical *C*(*t*) trajectories, so the cost is insensitive to which pattern is currently active. Apparent monotone decay of $J^{(k)}$ does not by itself imply convergence. The relative change diagnostic of Fig. 1 remains the authoritative test.

The failure rate scaling with γ is mechanistic, not bifurcation-driven. Through the state product factor $$\rho _2 = \gamma E S$$ in (31), larger γ pushes $$|v_2^{**}|$$ further outside the admissible interval and makes the support set $$\mathcal {S}_2^{(k)}$$ more sensitive to adjoint perturbations across iterations. The saddle-node bifurcation $$\textrm{SN}_2$$ at $$\gamma \approx 37.4167$$ (Zhang et al. 2024) is an independent property of the uncontrolled dynamics. The four sweep values are a biological grid rather than a bifurcation probe, and the same FBS failure would arise at any γ for which $$\rho _2 / \alpha _T$$ is large enough that the projected update saturates.

#### The damped regime converges at substantial cost

A practitioner attempting to fix the nominal-regime failure would typically reduce the damping factor and add adaptive damping. We implemented this fix and re-ran the same 4×4 grid with ω initialised at 0.1 and halved adaptively whenever the residual fails to decrease monotonically over a sliding window, with a floor at $\omega = 10^{-3}$ and an iteration cap of 500.

**Fig. 3:**
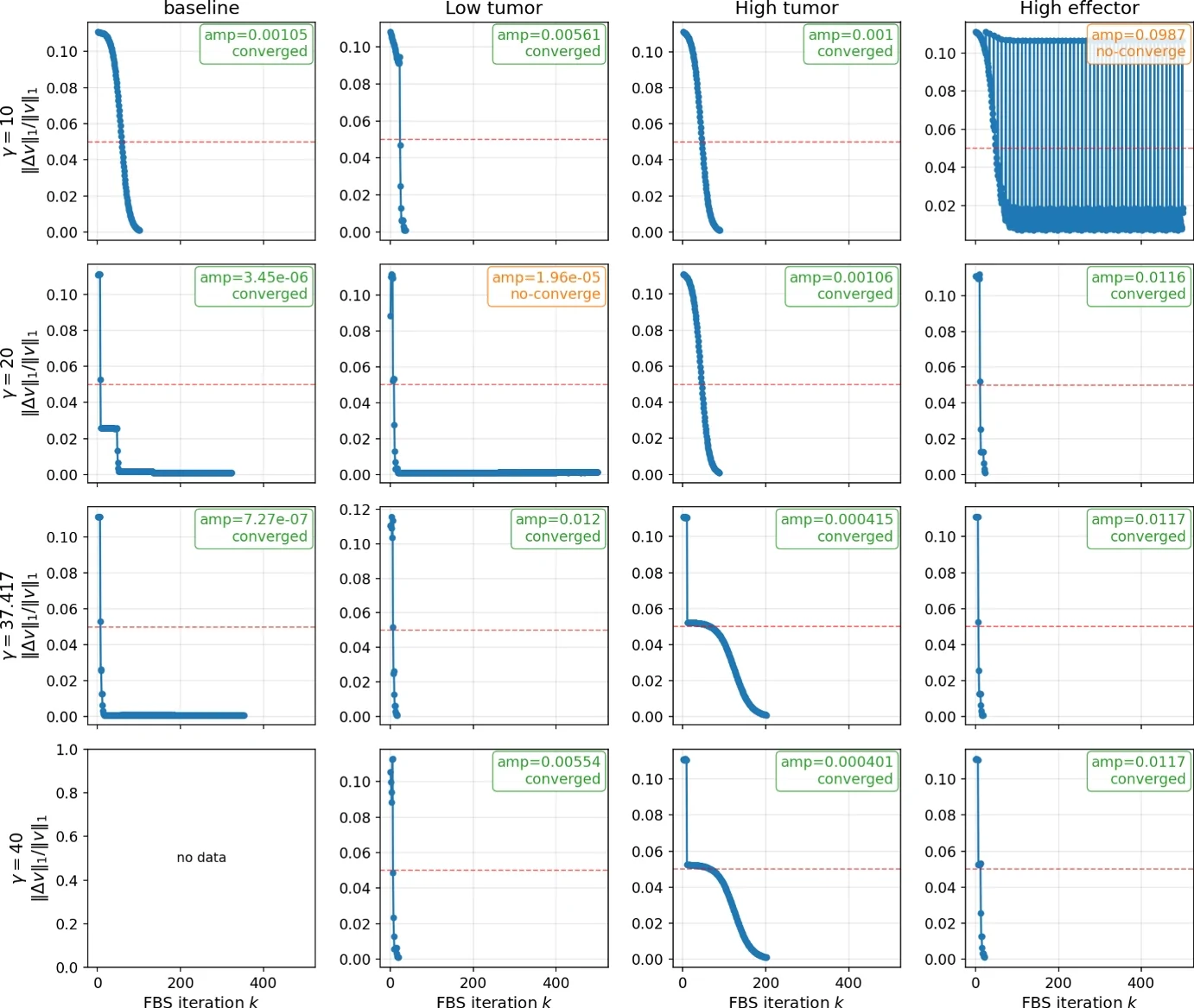
FBS relative change per iteration in the damped regime

With this remedy in place, FBS reaches a fixed point on 15 of the 16 scenarios, as shown in Fig. 3. Most scenarios now exhibit smooth monotone-decreasing residuals, in contrast to the saturated 2-cycles of the nominal regime. Among the 15 converged scenarios, 10 trigger the strict early-stop criterion $r^{(k)} < 10^{-3}$ at iteration counts ranging from 17 to 353. The remaining 5 hit the iteration cap of 500 with the residual already at the tolerance boundary $r^{(k)} \approx 10^{-3}$, effectively at a fixed point but without formally triggering early-stop. The worst converging scenario is the baseline IC in the delayed response regime at γ=37.417. It takes 353 iterations and approximately 87 minutes of single-core wallclock. The one remaining failure is the γ=40, baseline IC, which exceeds the 2-hour wallclock cap before completing. It is the empty panel in Fig. 3.

**Fig. 4:**
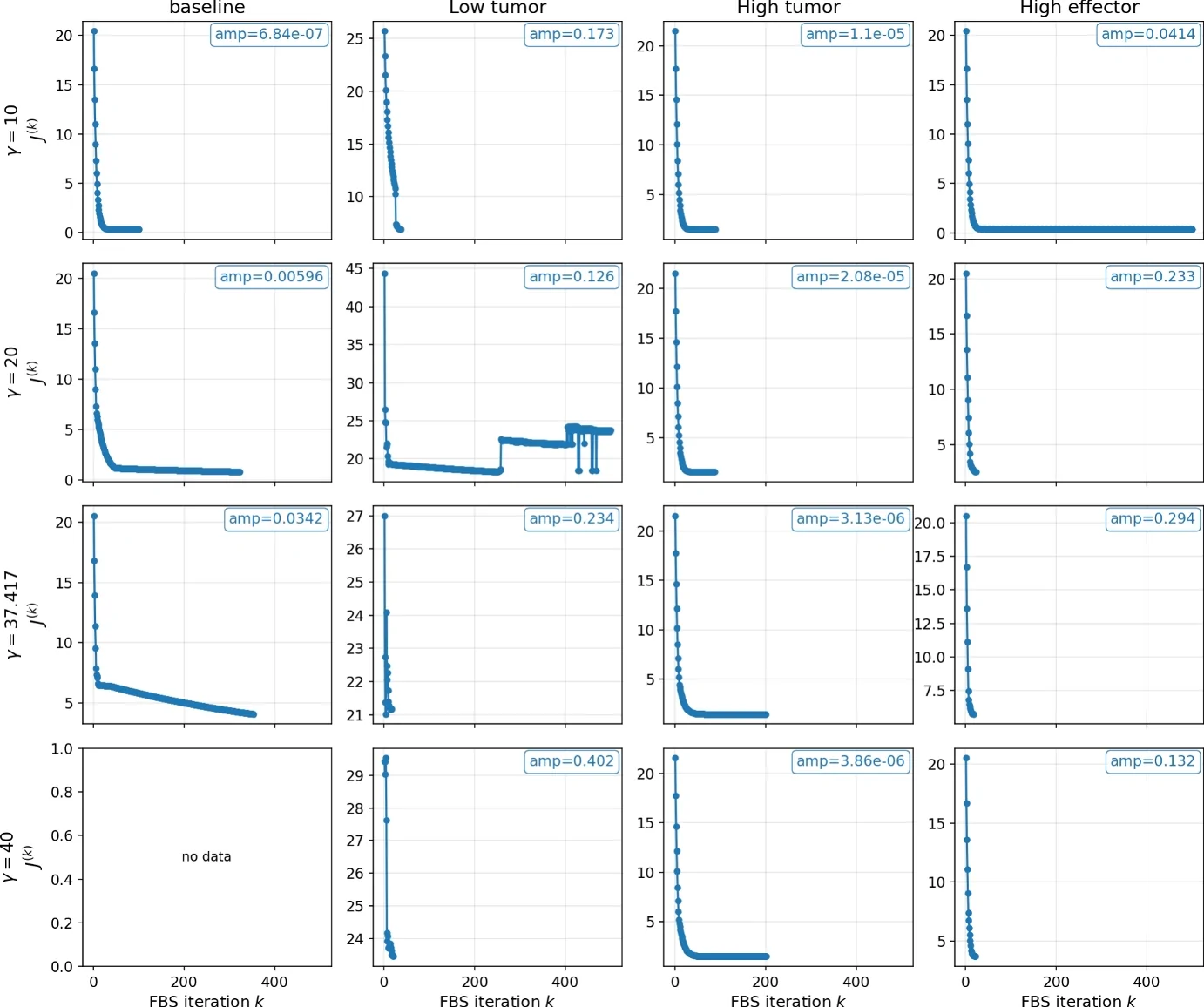
FBS objective $J^{(k)}$ across iterations in the damped regime

Figure 4 confirms the picture on the cost. $J^{(k)}$ now decreases monotonically in every converged scenario, in contrast to the wild oscillations of the nominal regime shown in Fig. 2. The damped regime fixes the convergence rate from 2/16 to 15/16. The cost is a 17× larger iteration cap, from 30 to 500 iterations, and a worst-case wallclock that grows from under two minutes to nearly an hour and a half. Even after this fix, one scenario still fails within a 2-hour budget. The full performance summary, including the benchmark against UDE, is given in Table 2 of Sect. 4.4. Each iteration over the OC formulation requires re-running this expensive computation. Examples include alternative cost weights, additional constraints, or extended controls such as the chemotherapy term in (37) of Sect. 4.6. We argue that this fragility is a structural property of the fixed point iteration rather than of the underlying optimal control problem.

### UDE backpropagation

We apply the UDE-based optimal control framework of Sect. 3, which is an instance of the universal differential equation formalism (Rackauckas et al. 2020), to the same 4×4 grid. The control is parameterised by a feed-forward neural network $\textrm{NN}_p$ with two hidden layers of width 16 and GELU activations (Hendrycks and Gimpel 2016), followed by an elementwise sigmoid that enforces $v_1(t), v_2(t) \in (0, 1)$.

34 $$\begin{aligned} \begin{aligned} \textrm{NN}_p(\cdot , \tau )&= W^{(3)}\, \textrm{GELU}\!\Bigl ( W^{(2)}\, \textrm{GELU}\!\bigl ( W^{(1)} \tau + b^{(1)} \bigr ) + b^{(2)} \Bigr ) + b^{(3)}, \\ v_i(t)&= \sigma \!\left( \textrm{NN}_p\bigl [\,i,\; \tau \bigr ] \right) , \qquad \tau = t / t_f, \quad i = 1, 2. \end{aligned} \end{aligned}$$

Here $\sigma (x) = 1/(1 + e^{-x})$ is the standard logistic sigmoid, applied elementwise. The parameter vector *p* collects $W^{(1)} \in \mathbb {R}^{16 \times 1}$, $W^{(2)} \in \mathbb {R}^{16 \times 16}$, $W^{(3)} \in \mathbb {R}^{2 \times 16}$, $b^{(1)}, b^{(2)} \in \mathbb {R}^{16}$, $b^{(3)} \in \mathbb {R}^{2}$, totalling 338 scalar parameters. The network is generated using the Julia package Lux.jl (Pal 2023a, b). Training proceeds on a uniform time grid of M=201 points. The schedule is 200 iterations of Adam (η=0.01) followed by 30 iterations of L-BFGS (Liu and Nocedal 1989; Fletcher 2000). Adjoint sensitivity is computed via the QuadratureAdjoint method (Rackauckas et al. 2020, §6.3), the recommended adjoint for small-state, large-parameter settings such as ours. ODE tolerances during training are $\textrm{rtol} = 10^{-6}$ and $\textrm{atol} = 10^{-7}$. The final evaluation uses tightened tolerances of $10^{-10}$.

**Fig. 5:**
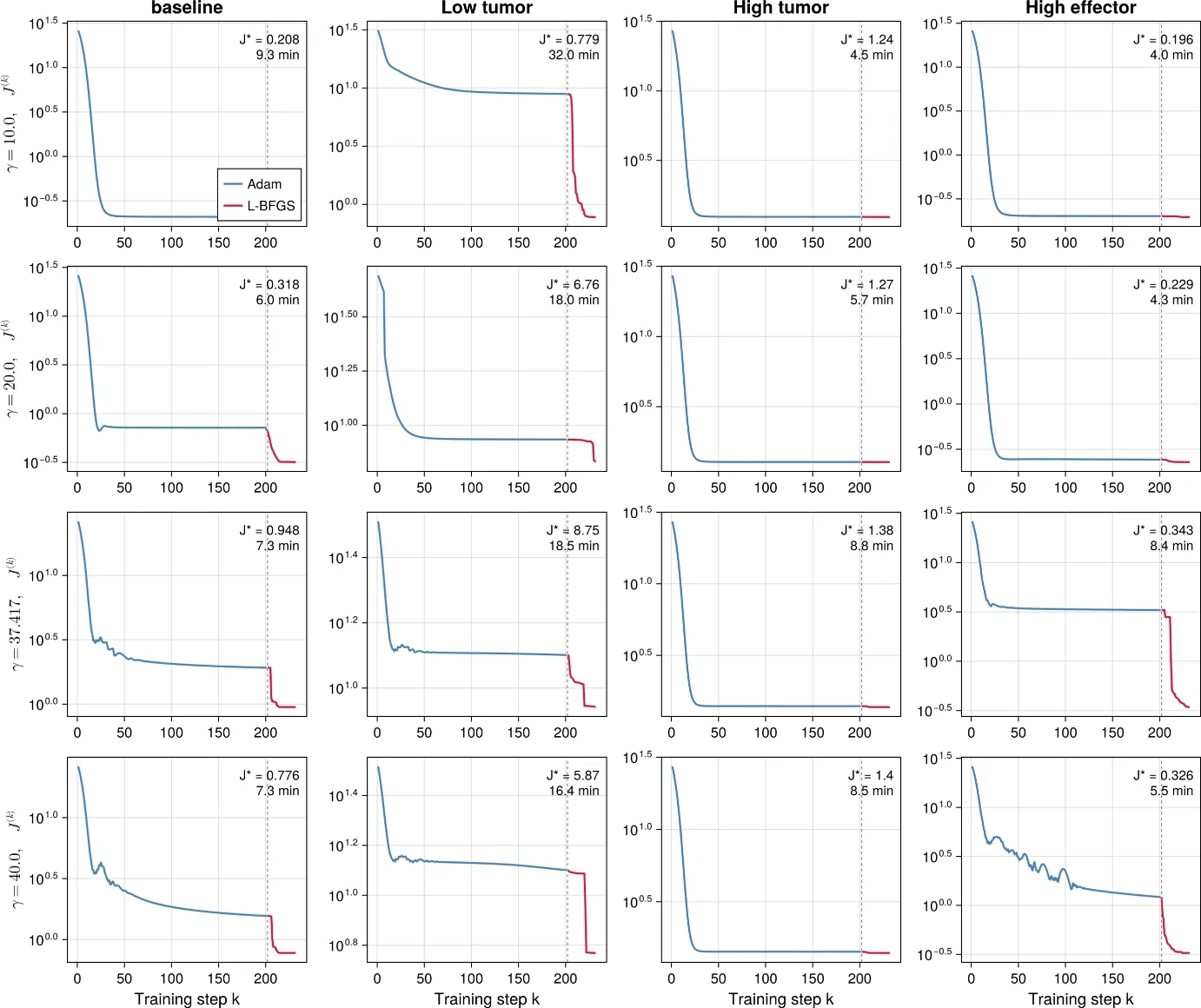
UDE training loss $J^{(k)}$ across the 4×4 grid

UDE has no fixed point operator and no projection step, so the FBS support set diagnostic of Sect. 4.3.1 does not apply. Convergence is defined directly through the loss trajectory. A training run has converged when $J^{(k)}$ decreases monotonically through the Adam phase and stabilises during the L-BFGS phase, with no oscillation between successive parameter updates. Because *J* is a smooth function of *p*, this monotone-then-stable behaviour is the gradient-descent analogue of the $r^{(k)} \rightarrow 0$ test in Eq. (32). All 16 scenarios satisfy the criterion. Figure 5 plots $J^{(k)}$ against training step *k* for every scenario, with rows indexing γ and columns indexing IC. The Adam phase (k=1,…,200) is shown in blue and the L-BFGS phase (k=201,…,230) in red, separated by a vertical dashed line at the handoff. The y-axis is log-scale, and per-scenario annotations report the final loss $J^{*}$ and the wallclock. In every panel the loss decreases monotonically through Adam and stabilises during L-BFGS. The per-scenario wallclock ranges from 4 to 32 minutes on a single laptop CPU core, with median ≈8 minutes. At the delayed response scenario (γ=37.417, baseline IC), $J^{(k)}$ decreases from $J^{(1)} = 26.32$ at random initialisation to J=1.93 at the end of Adam and to J=0.948 at the end of L-BFGS. Final losses across the grid range from $J^{*} = 0.20$ at baseline IC and high_effector scenarios, both with low to moderate tumor burden, to $J^{*} = 8.75$ at γ=37.417, low_tumor, where the integrated tumor burden is dominated by post-treatment regrowth.

The structural reason UDE avoids the FBS failure mode is the parameterisation. The control in (34) is a smooth function of the parameter vector *p* at every time point and across the entire grid. The logistic sigmoid saturates only asymptotically as the network weights grow large, and the adjoint method guarantees that the gradient $\partial J / \partial p$ is well-defined and accurately computable throughout training (Rackauckas et al. 2020). The three structural sources of FBS failure are therefore absent. First, there is no fixed point operator on the control as in the FBS update $v^{(k+1)} = \omega v^{*} + (1 - \omega ) v^{(k)}$ of Sect. 4.3. Second, there is no projection discontinuity at the box constraint boundary as in the $\max \{0, \min \{1, \cdot \}\}$ of Eq. (31). Third, there is no support set flip $\mathcal {S}_i^{(k)} \leftrightarrow \mathcal {S}_i^{(k+1)}$ of the kind that drove the sustained 2-cycle in Fig. 1. The control space is the smooth manifold of network parameters, on which gradient descent makes monotone progress.

Table 2 summarises the comparison on the 4×4 grid. Per-iteration cost is comparable, with one forward state ODE solve plus one backward adjoint ODE solve in both methods. The differential reliability lies in the iteration count distribution. FBS iteration counts vary sharply with the scenario, ranging from 17 to more than 500, and wallclocks from seconds to hours. UDE is uniform across the grid, taking 230 training iterations and 4 to 32 minutes per scenario.

**Table 2:** Comparison of nominal FBS, damped FBS, and UDE on the same 4×4 grid of $(\gamma , \textrm{IC})$ scenarios. UDE’s iteration cap of 230 is the fixed schedule of 200 Adam steps plus 30 L-BFGS steps, with no early stop tied to convergence. Wallclock numbers are single core on commodity hardware, FBS on AMD EPYC 7702 (Texas Tech University n.d.) and UDE on Apple Silicon, within a factor of two on per core throughput. Final-cost comparisons in the text use only the 15 scenarios where the damped sweep converged

	Nominal FBS	Damped FBS	UDE
Reached optimum / fixed point (over 16 scenarios)	2	15	16
Failed (sustained 2-cycle or timeout)	14	1	0
Iteration cap per scenario	30	500	230
Wallclock cap per scenario	none	2 hours	none
Median wallclock per scenario	<1 min	≈7 min	≈8 min
Worst-case wallclock per scenario	<2 min	≈87 min	≈32 min

### Biological interpretation of the trained optimal control

**Fig. 6:**
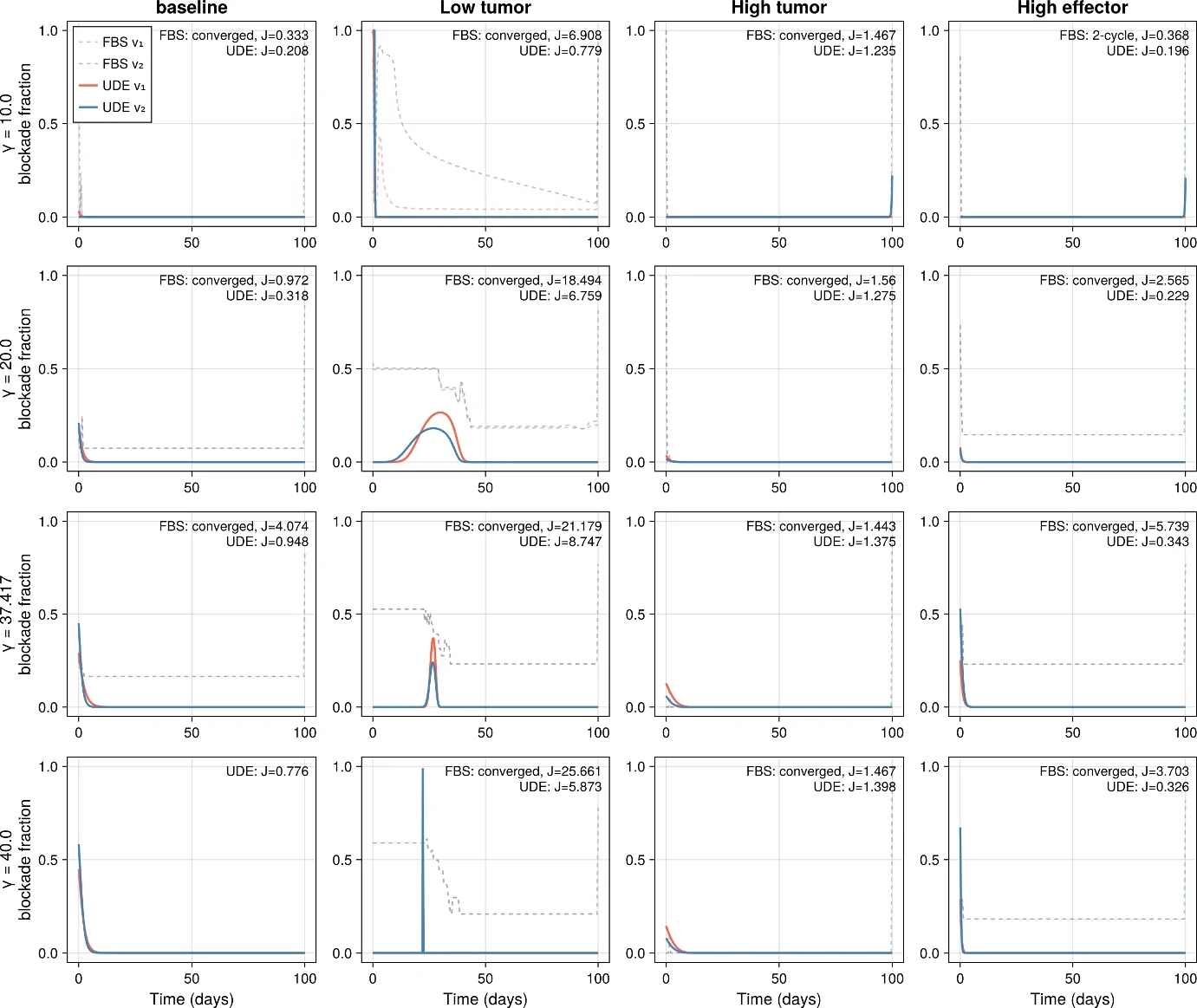
Damped-regime FBS converged iterates shown dashed versus UDE trained controls shown solid on the 4×4 grid

Figure 5 of Sect. 4.4 established that UDE training reaches a stable optimum on every scenario of the grid. We now examine the shape of the trained controls and read their biology. Figure 6 compares the damped-regime FBS converged iterate, shown dashed, against the UDE trained controls, shown solid, across the full 4×4 grid. Red curves show $$v_1(t)$$, the anti-CTLA-4 blockade fraction. Blue curves show $$v_2(t)$$, the anti-PD-1 blockade fraction. Per-scenario annotations report the FBS verdict and the final cost $$J^{*}$$ for each method. UDE produces smooth, monotone controls in every scenario, typically a transient pulse over the first 10 to 30 days that decays to zero. The damped FBS converged iterate is piecewise constant at saturation levels over most of the time grid, with step transitions where the projection clips $$v_i^{**}$$ from (33) to [0, 1]. Most cells also exhibit spikes at $$t = t_f$$ driven by the transversality $$\lambda _4(t_f) = -\psi _E$$. The contrast with UDE is sharpest at γ=37.417, low_tumor, where UDE produces a clean bell-shaped pulse around day 25 while the FBS iterate switches abruptly between 0 and a plateau near 0.55 across the same window. The smoothness of the UDE controls makes them directly readable as continuous dosing schedules. The step changes of the FBS solution are not realisable as clinical infusion rates. The γ=40, baseline IC panel has no FBS data because that damped-regime run exceeded the 2-hour wallclock cap.

The annotations also let us compare final costs. We make this comparison only on the 15 scenarios where the damped sweep reached a converged fixed point. We never compare against the nominal sweep, whose saturated 2-cycle never settles to a single objective value, so the comparison does not rest on an unconverged cost. The comparison is therefore between two converged and feasible controls. The converged damped FBS iterate and the UDE control both satisfy the admissibility constraint $v_i \in [0, 1]$. Each is a feasible solution of the same optimal control problem with the same cost functional. Each therefore has a well-defined cost, and the two $J^{*}$ values are directly comparable. On these scenarios UDE consistently reaches a lower $J^{*}$ than the converged damped FBS iterate, typically by a factor of 2 to 5, and by more than 10 in several high-effector and high-γ scenarios. The discrepancy reflects that FBS converges to a piecewise-constant Pontryagin stationary point under the box constraint, while UDE searches a smooth control manifold and reaches a different local optimum with lower cost.

At the delayed response scenario with γ=37.417 and baseline IC (Zheng and Kim 2021), the trained UDE control is a burst-then-withdraw regime. A moderate dose pulse of combined co-stimulation and checkpoint blockade therapy in the first ≈10 days drives the cancer population from $C(0) = 10^3$ to C≈0 on the same timescale. Both controls then decay smoothly to zero, and treatment is withheld for the remaining ≈90 days. The optimiser correctly trades continued dosing for the toxicity penalty once C→0, because the cost functional has no explicit reward for sustained *E*(*t*) after eradication. The next section reformulates the cost functional to recover an induction maintenance schedule that prevents this recurrence, alongside two further cost functional variants.

### Multiple OC formulations on the same biological model

A practical advantage of the UDE pipeline is that it allows the optimal control problem to be modified at the cost functional level with minimal pipeline change. We exploit this here to study three OC variants of the cancer immunotherapy model at the delayed response scenario with γ=37.417 and baseline IC (Zheng and Kim 2021). Each variant addresses a known limitation of the canonical formulation (30) discussed in Sect. 4.5.

The first variant, called induction maintenance, addresses the lack of a maintenance phase in the canonical burst-then-withdraw schedule. To recover an induction maintenance pattern more representative of clinical practice (Larkin et al. 2015), we add a running penalty on the tumor population *C*(*t*) over the post-induction window $[t_{\text {ind}}, t_f]$,

35 $$\begin{aligned} J_{\text {indmaint}}(v_1, v_2) \!=\! \int _0^{t_f}\!\!\Bigl [\alpha _C C + \tfrac{\alpha _T}{2}(v_1^2 + v_2^2)\Bigr ] dt \!+\! \psi _C^{\text {run}} \!\!\int _{t_{\text {ind}}}^{t_f}\!\! C(t)\, dt \!+\! \psi _C C(t_f) - \psi _E E(t_f), \end{aligned}$$

with $t_{\text {ind}} = 30$ days and recurrence-penalty weight $\psi _C^{\text {run}} = 50$. The state equations are unchanged. The penalty directly punishes any regrowth of *C*(*t*) in the maintenance window. It is the cost functional analogue of the clinical motivation for monotherapy maintenance, which is that cancer should not be allowed to recur after induction.

The second variant, called toxicity budget, treats cumulative dose toxicity as a hard upper bound rather than a running penalty. The canonical cost penalises toxicity through the running term $\frac{\alpha _T}{2}(v_1^2 + v_2^2)$. We replace this running toxicity term with a soft Lagrangian penalty for exceeding a fixed cumulative dose squared budget $B_{\text {tox}}$,

36 $$\begin{aligned} J_{\text {toxbudget}}(v_1, v_2) = \int _0^{t_f}\!\!\alpha _C C(t)\,dt + \lambda _{\text {pen}}\,\bigl [\max (0,\, T - B_{\text {tox}})\bigr ]^2 + \psi _C C(t_f) - \psi _E E(t_f), \end{aligned}$$

where $T = \int _0^{t_f}(v_1^2 + v_2^2)\,dt$ is the realised cumulative dose squared. We use $B_{\text {tox}} = 0.3$, a tight budget below the canonical variant realised toxicity, and $\lambda _{\text {pen}} = 100$.

The third variant, chemo plus immunotherapy, extends the control set to include chemotherapy by introducing a third blockade fraction $v_3(t) \in [0, 1]$ representing chemotherapy receptor occupancy. The cancer-growth equation gains a multiplier $u_3 = 1 - \alpha _3 v_3$ that reduces the effective growth rate when chemotherapy is administered, with drug class efficacy ceiling $\alpha _3 = 1$. The state equations become

37 $$\begin{aligned} \begin{aligned} \dot{C}&= u_3(t)\, F(C)\, C - \kappa \, C\, E, \\ \dot{A}&= r_A C - \delta _A A, \quad \dot{I} = r_I C E - \delta _I I, \\ \dot{E}&= -r_E(E - E^{*}) + (1 + \alpha _1 v_1)\beta A I E S - (1 - \alpha _2 v_2)\gamma E S, \\ \dot{S}&= -r_S(S - S^{*}) - (1 + \alpha _1 v_1)\beta A I E S + (1 - \alpha _2 v_2)\gamma E S. \end{aligned} \end{aligned}$$

The cost functional adds a third quadratic toxicity term but with a *separate* toxicity weight $\alpha _T^{\text {chemo}}$ on $v_3$. This reflects that chemotherapy and immunotherapy have qualitatively different adverse event profiles and therefore different per-dose toxicity costs. The cost functional is

38 $$\begin{aligned} J_{\text {chemoimmu}}(v_1, v_2, v_3) \!=\! \int _0^{t_f}\!\!\Bigl [\alpha _C C + \tfrac{\alpha _T}{2}(v_1^2 + v_2^2) \!+\! \tfrac{\alpha _T^{\text {chemo}}}{2} v_3^2\Bigr ] dt \!+\! \psi _C C(t_f) - \psi _E E(t_f). \end{aligned}$$

We use $\alpha _T^{\text {chemo}} = 0.3$, reflecting clinical experience that immune-related adverse events scale more aggressively with dose than chemotherapy class adverse events do per unit receptor occupancy. The network output layer has three sigmoid neurons producing $(v_1, v_2, v_3)$ directly.

*Pipeline reuse* is the methodological point of this section. Each of the three variants is trained with the same UDE pipeline of Sect. 4.4, the same 200-iteration Adam plus 30-iteration L-BFGS schedule, and the same QuadratureAdjoint sensitivity computation. The only changes are the loss-function definition in Variants 1 and 2 and the network output dimension plus state equation modification in Variant 3. The training scripts CancerImmu_indmaint.jl, CancerImmu_toxbudget.jl, and CancerImmu_chemoimmu.jl are derived from the canonical script by these focused changes.

**Fig. 7:**
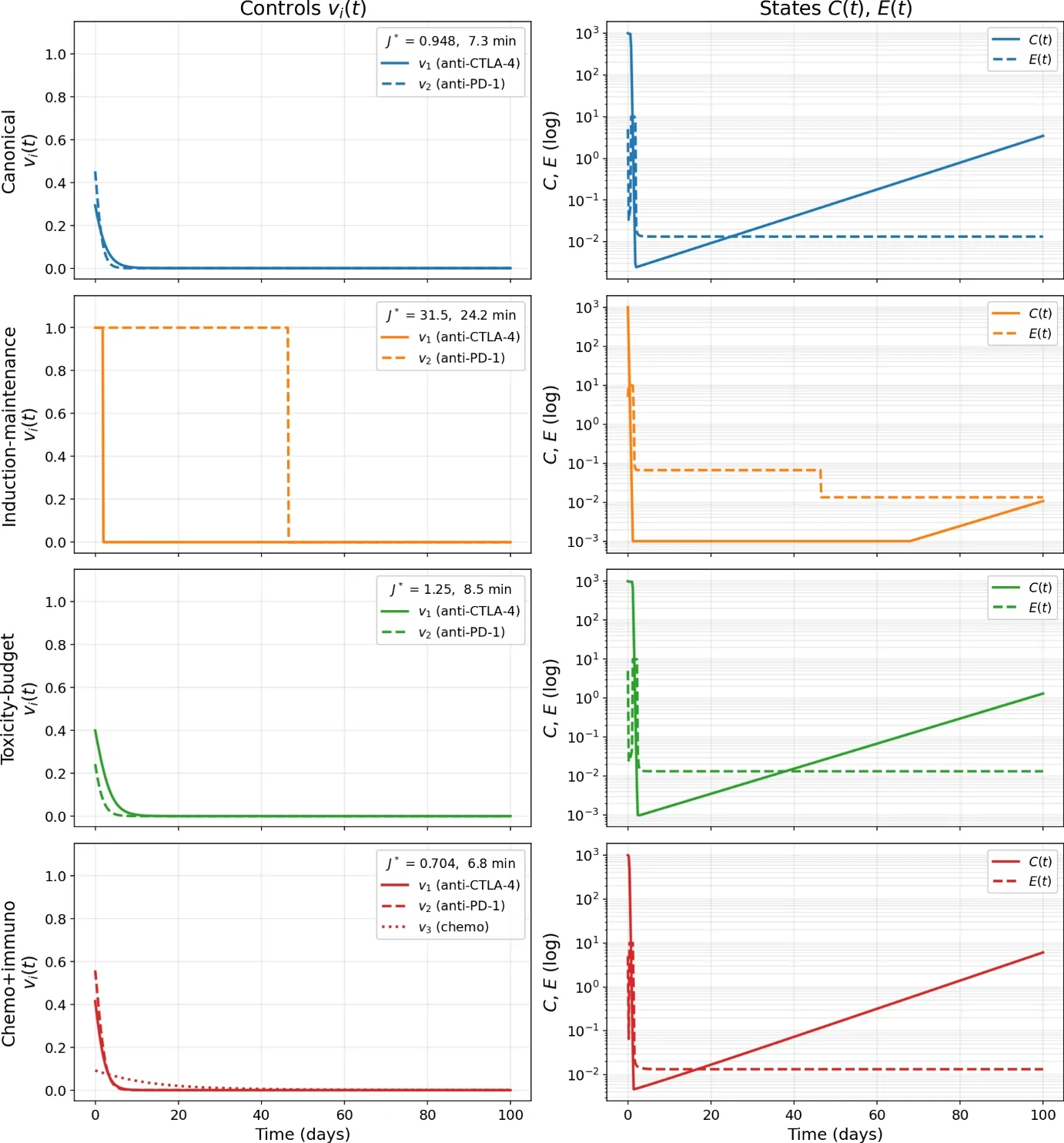
Trained UDE controls and state trajectories per variant

As shown in Fig. 7, the four trained schedules are qualitatively different. The induction maintenance variant saturates $v_2 = 1$ throughout the first ≈45 days, then withdraws abruptly. Under this sustained anti-PD-1 dosing the tumor population stays at $C \approx 10^{-3}$ for roughly 70 days, four decades below the canonical and chemo+immuno trajectories at the same time points. The effector population is maintained at E≈0.07, about five times the suppressed-immune attractor value. The toxicity budget variant produces a smaller initial pulse with $v_2(0) \approx 0.25$, compared to ≈0.45 in the canonical case, and modestly higher $v_1(0)$, achieving cancer eradication at substantially lower cumulative dose. The chemo+immuno variant uses chemotherapy modestly, with $v_3(0) \approx 0.09$ decaying by day 30, on top of an immunotherapy schedule similar to the toxicity budget case. Chemo does not substitute for immunotherapy because the kill term κCE depends on effector cells *E*, not on chemotherapy, so chemo can only complement the immune kill mechanism by halting growth, not replace it.

The $J^{*}$ values reported in Fig. 7 are not directly comparable across variants because each variant uses a different cost functional. In particular, the induction maintenance value $J^{*}_{\text {indmaint}} = 31.5$ is dominated by the recurrence-penalty contribution $\psi _C^{\text {run}} \int _{30}^{100}\! C(t)\,dt \approx 30$, which is exactly the term the optimiser was given to minimise. The remaining canonical-style cost terms are of the same magnitude as $J^{*}_{\text {canonical}} = 0.948$. Despite the largest $J^{*}$, induction maintenance achieves the strongest underlying tumor control performance, with a 4-decade suppression of *C*(*t*) sustained over 70 days. The wallclock values shown in Fig. 7 all fall in the range 7 to 25 minutes, illustrating that pipeline reuse comes at no significant per-scenario compute cost.

The biological interpretation is that the cost functional change determines which clinical knob the optimiser reaches for. The recurrence penalty asks the optimiser to suppress *C*(*t*) during the maintenance window and the optimiser does so by sustaining $v_2$, matching the induction maintenance pattern of real clinical practice (Larkin et al. 2015). The hard toxicity budget asks the optimiser to ration cumulative dose and it does so by lowering $v_2$ while modestly increasing $v_1$. The added chemotherapy channel is used as a complement to immunotherapy rather than a substitute, because the model’s kill term depends on effector cells alone. Each result is a faithful image of the cost functional and the model.

**Table 3:** Sobol indices for the four UDE cost weights

	$\alpha _C$		$\alpha _T$		$\psi _C$		$\psi _E$	
Output	$S_i$	$S_{T_i}$	$S_i$	$S_{T_i}$	$S_i$	$S_{T_i}$	$S_i$	$S_{T_i}$
$J^{*}$	0.00	0.00	0.39	$\mathbf {0.88}$	0.00	0.47	0.08	0.48
$\textrm{AUC}_C$	0.02	0.24	0.00	0.00	0.00	0.61	0.22	$\mathbf {0.93}$
$\textrm{AUC}_{v_1^2}$	0.05	0.07	0.12	0.16	0.13	$\mathbf {0.74}$	0.00	0.63
$\textrm{AUC}_{v_2^2}$	0.27	0.37	0.32	$\mathbf {0.56}$	0.05	0.18	0.12	0.29
$E(t_f)$	0.00	0.00	0.00	0.00	0.29	$\mathbf {0.95}$	0.10	0.76

Bold entry in a row indicates the dominant input for that output. If you want to predict or control that output, pin down this input first

### Sensitivity analysis on the cost weights

The canonical cost weights $(\alpha _C, \alpha _T, \psi _C, \psi _E) = (10^{-3}, 1, 10^{-2}, 1)$ were chosen by scale-balancing, not by calibration to any clinical outcome. A natural question is how robust the trained schedule properties are to perturbations of these weights. The standard tool is a global sensitivity analysis based on Sobol indices (Sobol’ 2001), which decomposes the variance of a model output across variations in each input and all of their interactions. The challenge is the expensive nature of the underlying model. Each model evaluation here is one UDE training at one cost weight vector, which takes about 7 to 10 minutes of wallclock. Direct application of the Sobol estimator on the UDE would require $\sim 10^4$ such evaluations, roughly 60 days of compute. Surrogate-based estimation (Forrester et al. 2008) replaces the expensive model with a cheap statistical approximation, a Gaussian process, trained from a small number of expensive samples and evaluated many times.

We draw N=40 Latin hypercube samples (McKay et al. 1979) from the cube $[0.5\times , 1.5\times ]^4$, in which each weight is rescaled from 0.5 to 1.5 times its baseline value. At each sample we train the canonical UDE at γ=37.417 and baseline IC by the pipeline of Sect. 4.4, ≈5.3 hours of wallclock in total. From each trained controller we extract by trapezoidal integration the final loss $J^{*}$, the cumulative tumor burden $\textrm{AUC}_C = \int _0^{t_f} C(t)\,dt$, the cumulative dose squared toxicities $\textrm{AUC}_{v_1^2} = \int _0^{t_f} v_1(t)^2\,dt$ and $\textrm{AUC}_{v_2^2} = \int _0^{t_f} v_2(t)^2\,dt$, and the terminal effector population $E(t_f)$. One Gaussian process surrogate (Rasmussen and Williams 2006) per output is fit in scikit-learn (Pedregosa et al. 2011) with an anisotropic Matérn ν=5/2 kernel plus white noise, hyperparameters tuned by log marginal likelihood. SALib (Saltelli et al. 2010) then draws a Saltelli matrix of N(D+2)=6144 points at base N=1024, the surrogates predict at every point, and the SALib Sobol estimator returns first-order $S_i$ and total-order $S_{T_i}$ Sobol indices (Sobol’ 2001) with bootstrapped 95% confidence intervals.

**Fig. 8:**
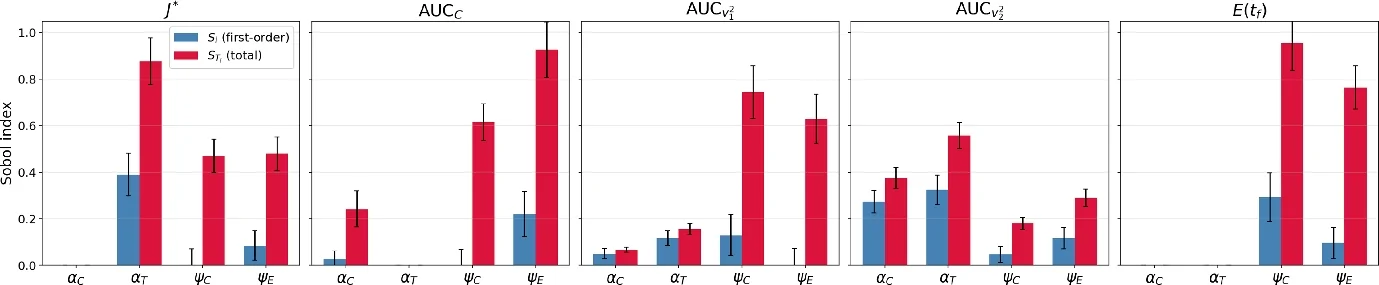
Sobol indices $S_i$ and $S_{T_i}$ for the four UDE cost weights, one panel per scalar output. Error bars are bootstrapped 95% confidence intervals

Sobol indices for the four UDE cost weights on the five scalar outputs are reported in Table 3 and Fig. 8. Both first-order $S_i$ and total-order $S_{T_i}$ are shown side by side. The first-order index $S_i$ is the share of output variance driven by weight *i* acting alone, while the total-order index $S_{T_i}$ also includes all interactions of weight *i* with the others. By construction $S_{T_i} \ge S_i$, and a large gap signals that the weight matters mostly through interactions. Bootstrap 95% confidence intervals are typically ±0.05 to ±0.12 on $S_{T_i}$. Both indices were estimated on a Gaussian process surrogate trained on N=40 LHS samples in the perturbation cube $[0.5\times , 1.5\times ]^4$ around the baseline weights, at the canonical scenario γ=37.417 and baseline IC. Total-order indices are large for at least one weight per output, but no single weight dominates all five outputs.

Reading down Table 3, three concrete findings drop out. First, the toxicity weight $\alpha _T$ is the dominant driver of the trained loss $J^{*}$ at $S_T = 0.88$. It is the cost parameter most worth pinning down with clinical adverse event data in any follow-up calibration. Second, the cumulative tumor exposure $\textrm{AUC}_C$ is dominated by the terminal effector reward $\psi _E$ at $S_T = 0.93$, which sets how aggressively the schedule pushes *C*(*t*) down over the horizon. The more we reward retaining *E*(*t*) at $t_f$, the more rapid the tumor kill. Third, the terminal effector $E(t_f)$ is almost entirely set by the terminal tumor weight $\psi _C$ at $S_T = 0.95$. The trade-off between $\psi _C$ and $\psi _E$ in the terminal cost expression $\psi _C C(t_f) - \psi _E E(t_f)$ determines the asymptotic immune state. The running tumor weight $\alpha _C$ has the smallest total-order effect across all five outputs, with $\max S_T \approx 0.37$. Within the $[0.5\times , 1.5\times ]$ range it modulates magnitude rather than the shape of the trained schedule.

In Fig. 8, the substantial gap $S_{T_i} - S_i$ on several outputs, notably $\textrm{AUC}_{v_1^2}$ and $E(t_f)$, shows that the cost weight effects are not separable. The optimal control schedule responds to combinations of weights, not to any one weight in isolation. This is consistent with the cost functional structure of Eq. (30), where the running and terminal terms couple through the state trajectory.

The trained schedule has a burst-then-withdraw shape, with cancer eradicated within ∼10 days and the state relaxing afterward to the suppressed immune attractor. This shape is preserved across all 40 LHS samples in the $[0.5\times , 1.5\times ]$ perturbation range. Weight perturbations of this magnitude rescale the schedule’s amplitude and timing but leave its structural shape intact. The methodological conclusions of Sects. 4.4 through 4.6 are therefore robust to weight perturbations of this magnitude. These are UDE pipeline reliability, flexibility across cost formulations, and a schedule shape that changes qualitatively only when the cost functional itself is changed.

## Conclusion and discussion

This paper frames the optimal control problem within the UDE framework and trains a neural network to approximate the control variables. The first-order necessary conditions of Pontryagin’s Maximum Principle are met at any stationary point of the training loss, up to a discretisation-controlled accuracy.

We apply the framework to the immune-cancer dynamics model (Zheng and Kim 2021) on a 4×4 grid of bifurcation parameter and initial condition scenarios. The standard forward-backward sweep method fails on 14 of 16 scenarios in the nominal regime by a saturated 2-cycle of the FBS iterates, documented in Sect. 4.3.1. UDE training reaches a stable optimum on every scenario, lowers $J^{*}$ by a factor of 2 to 10 relative to the converged damped FBS iterate, and produces smooth time-varying controls that can be programmed directly as a continuous drug infusion schedule. Cost functional variants are accommodated by changing the loss definition with no change to the training pipeline. A Sobol global sensitivity analysis on the four cost weights confirms that the burst-then-withdraw schedule shape is preserved across 40 Latin hypercube samples in the $[0.5\times , 1.5\times ]^4$ perturbation cube.

Beyond cancer therapy, the framework may extend to other biological optimal control problems with bounded controls, stiff dynamics, and a saturating optimum. Candidate applications include HIV optimal treatment, vaccination control on epidemic models, and harvesting control in ecology. We have not validated these extensions in the present work.

The contributions of this paper are bounded by five formulation and scope choices.

First, the toxicity term in the cost functional is a quadratic penalty $\tfrac{\alpha _T}{2}(v_1^2 + v_2^2)$ on the blockade fractions, chosen for convexity in *v*, agreement with the linear-quadratic dose-response model of radiation oncology (Withers 1975; Brenner 2008), and tractability. It does not capture threshold-style immune-related adverse events at high checkpoint inhibitor doses (Naidoo et al. 2015). A Hill-saturating penalty $c\, v^2 / (K + v^2)$ is the natural extension, with *c* and *K* calibrated from adverse-event data.

Second, the cost weights $\alpha _C, \alpha _T, \psi _C, \psi _E$ are scale-balanced rather than calibrated to clinical quality-adjusted life year data (Lenhart and Workman 2007). Section 4.7 confirms that the qualitative trained schedule shape is preserved across 40 Latin hypercube samples in the $[0.5\times , 1.5\times ]^4$ perturbation cube. The toxicity weight $\alpha _T$ and the terminal effector reward $\psi _E$ are the two weights most worth pinning down in any follow-up clinical calibration. A related feature is that the cost has no reward for sustained *E*(*t*) after C→0, so the trained schedule withdraws to zero after eradication rather than continuing maintenance dosing. The induction maintenance variant of Sect. 4.6 addresses this directly.

Third, the entire study uses a single biological model (Zheng and Kim 2021). We document a specific structural FBS failure mode on this model, namely a saturated 2-cycle of the FBS iterates. Whether the same mechanism arises on other biological optimal control problems is a follow-up question.

Fourth, UDE training reaches a local stationary point of the loss, not a global optimum. Theorem 1 guarantees the PMP first-order necessary conditions at any such stationary point, but global optimality is not. A multi-start sweep over random seeds would characterise the empirical $J^{*}$ distribution. A deep BSDE solver for the Hamilton-Jacobi-Bellman equation (Han et al. 2018) would give a true global $J^{*}$ for comparison and is computationally feasible at the 5-dimensional state of our model.

Fifth, the comparison is to forward-backward sweep only. Direct collocation (Betts 2010), indirect semi-discretisation (Hager 2000), and differential dynamic programming (Mayne 1966) each have their own failure modes, and a fair multi-method comparison would constitute a separate study.

Two follow-up directions arise naturally. The first is a method survey applying the UDE pipeline to other mathematical-biology optimal control problems, including HIV optimal treatment, vaccination control on epidemic models, and harvesting control in ecology. The second is a clinical-grade extension of the cancer immunotherapy application, calibrating cost weights from QALY data, fitting Hill-saturating toxicity penalties from adverse-event datasets, and comparing the trained schedules to established clinical protocols.

**Table 4:** Summary of baseline parameter estimates (Zheng and Kim 2021)

Symbol	Description	Estimate
$r_C$	Logistic growth rate of cancer	$\hbox {1.0 day}^{-1}$
$r_{\text {max}}$	Maximum growth rate of cancer	$\hbox {0.09 day}^{-1}$
$C^*$	Cancer steady state concentration	$10^3$ cells/nL
κ	Killing rate of cancer cells by T cells	1.2 nL/(cells·day)
$r_A$	Antigen presentation source rate	0.5 pep/(nL·day)
$\delta _A$	Antigen presentation degradation rate	$\hbox {0.8 day}^{-1}$
$r_I$	Inflammation source rate	0.4 (ng·nL)/($\hbox {cells}^2\cdot$day)
$\delta _I$	Inflammation degradation rate	$\hbox {3.0 day}^{-1}$
$r_E$	Effector T cell growth coefficient	$\hbox {1.0 day}^{-1}$
$E^*$	Effector T cell base steady state	5.0 cells/nL
$r_S$	Non-effector T cell growth coefficient	$\hbox {1.0 day}^{-1}$
$S^*$	Non-effector T cell base steady state	5.0 cells/nL
β	Effector T cell recruitment coefficient	$\hbox {0.009 nL}^3$/(pep·ng·cells·day)
γ	Non-effector T cell recruitment coefficient	37.417 nL/(cells·day)

## Data Availability

No external datasets were generated or analysed during this study. All numerical inputs are specified in the manuscript.

## Appendix A Pontryagin necessary conditions for the proposed reformulation

For the state system (28) with the controls in (29) and the cost (30), introduce the adjoint vector $\lambda (t) = (\lambda _1, \ldots , \lambda _5)^{\top }$. The Hamiltonian is

A1 $$\begin{aligned} \begin{aligned} H(y, v, \lambda )&= \alpha _C\, C + \tfrac{\alpha _T}{2}(v_1^2 + v_2^2) + \lambda _1\bigl [F(C)C - \kappa C E\bigr ] + \lambda _2\bigl [r_A C - \delta _A A\bigr ] \\&\quad + \lambda _3\bigl [r_I C E - \delta _I I\bigr ] + \lambda _4\bigl [-r_E(E - E^{*}) + (1 + \alpha _1 v_1)\beta A I E S - (1 - \alpha _2 v_2)\gamma E S\bigr ] \\&\quad + \lambda _5\bigl [-r_S(S - S^{*}) - (1 + \alpha _1 v_1)\beta A I E S + (1 - \alpha _2 v_2)\gamma E S\bigr ]. \end{aligned} \end{aligned}$$

With $F^{\prime }\left(C\right):=d\left[F\left(C\right)C\right]/dC$ equal to $$r_{\max }$$ on the saturated branch and $$r_C(1 - 2C/C^{*})$$ otherwise, applying $$\dot{\lambda }_i = -\partial H / \partial y_i$$ gives the adjoint system

A2 $$\begin{aligned} \dot{\lambda }_1= &  -\Bigl [\alpha _C + \lambda _1\bigl (F'(C) - \kappa E\bigr ) + \lambda _2 r_A + \lambda _3 r_I E\Bigr ], \nonumber \\ \dot{\lambda }_2= &  \lambda _2 \delta _A - (\lambda _4 - \lambda _5)(1 + \alpha _1 v_1)\beta \, I E S, \nonumber \\ \dot{\lambda }_3= &  \lambda _3 \delta _I - (\lambda _4 - \lambda _5)(1 + \alpha _1 v_1)\beta \, A E S, \nonumber \\ \dot{\lambda }_4= &  \lambda _1 \kappa C - \lambda _3 r_I C + \lambda _4 r_E - (\lambda _4 - \lambda _5)\bigl [(1 + \alpha _1 v_1)\beta \, A I S - (1 - \alpha _2 v_2)\gamma \, S\bigr ], \nonumber \\ \dot{\lambda }_5= &  \lambda _5 r_S - (\lambda _4 - \lambda _5)\bigl [(1 + \alpha _1 v_1)\beta \, A I E - (1 - \alpha _2 v_2)\gamma \, E\bigr ], \end{aligned}$$

with terminal conditions $\lambda (t_f) = \nabla \phi (y(t_f)) = (\psi _C,\, 0,\, 0,\, -\psi _E,\, 0)^{\top }$. The Hamiltonian is strictly convex in *v*, so $\partial H / \partial v_i = 0$ has a unique unconstrained minimiser, and elementwise projection onto the admissible set yields the optimal controls

A3 $$\begin{aligned} \begin{aligned} v_1^{*}(t)&= \max \!\Bigl \{0,\, \min \!\Bigl \{1,\, \tfrac{(\lambda _5 - \lambda _4)\,\alpha _1\,\beta \, A I E S}{\alpha _T}\Bigr \}\Bigr \}, \\ v_2^{*}(t)&= \max \!\Bigl \{0,\, \min \!\Bigl \{1,\, \tfrac{(\lambda _5 - \lambda _4)\,\alpha _2\,\gamma \, E S}{\alpha _T}\Bigr \}\Bigr \}, \end{aligned} \end{aligned}$$

all evaluated along the optimal trajectory $(y^{*}, \lambda )$. This is the form of the optimality conditions in Eq. (31) of the main text. The transversality has $\lambda _5 - \lambda _4 = \psi _E > 0$ at $t = t_f$, so the unconstrained stationary point of $v_i^*$ at $t_f$ is non-zero. Whether the constrained optimum then exhibits terminal blockade depends on the magnitude of the unconstrained value relative to the box [0, 1].

## Code availability

The Julia training scripts and the forward-backward sweep benchmark code are available from the corresponding author on reasonable request.
